# Cell-to-Cell Transmission Can Overcome Multiple Donor and Target Cell Barriers Imposed on Cell-Free HIV

**DOI:** 10.1371/journal.pone.0053138

**Published:** 2013-01-07

**Authors:** Peng Zhong, Luis M. Agosto, Anna Ilinskaya, Batsukh Dorjbal, Rosaline Truong, David Derse, Pradeep D. Uchil, Gisela Heidecker, Walther Mothes

**Affiliations:** 1 Department of Microbial Pathogenesis, Yale University School of Medicine, New Haven, Connecticut, United States of America; 2 HIV Drug Resistance Program, National Cancer Institute-Frederick, Frederick, Maryland, United States of America; INSERM, France

## Abstract

Virus transmission can occur either by a cell-free mode through the extracellular space or by cell-to-cell transmission involving direct cell-to-cell contact. The factors that determine whether a virus spreads by either pathway are poorly understood. Here, we assessed the relative contribution of cell-free and cell-to-cell transmission to the spreading of the human immunodeficiency virus (HIV). We demonstrate that HIV can spread by a cell-free pathway if all the steps of the viral replication cycle are efficiently supported in highly permissive cells. However, when the cell-free path was systematically hindered at various steps, HIV transmission became contact-dependent. Cell-to-cell transmission overcame barriers introduced in the donor cell at the level of gene expression and surface retention by the restriction factor tetherin. Moreover, neutralizing antibodies that efficiently inhibit cell-free HIV were less effective against cell-to-cell transmitted virus. HIV cell-to-cell transmission also efficiently infected target T cells that were relatively poorly susceptible to cell-free HIV. Importantly, we demonstrate that the donor and target cell types influence critically the extent by which cell-to-cell transmission can overcome each barrier. Mechanistically, cell-to-cell transmission promoted HIV spread to more cells and infected target cells with a higher proviral content than observed for cell-free virus. Our data demonstrate that the frequently observed contact-dependent spread of HIV is the result of specific features in donor and target cell types, thus offering an explanation for conflicting reports on the extent of cell-to-cell transmission of HIV.

## Introduction

Viruses can spread either by a cell-free mode through the extracellular space or by cell-to-cell transmission through direct cell-cell contact [1], [2], [3], [4]. For many viruses, preferences for either pathway have been known for many years. Many bacteriophages and some Alphaviruses are highly infectious in their cell-free form and a single viral particle can enter a cell and cause an infection [5], [6]. If these viruses also use cell-cell contact to spread is unknown. In contrast, the infectivity to particle ratio of other viruses can be very poor despite the observation of efficient spreading in tissue cultures [7], [8], [9]. This observation prompted the study of cell-to-cell transmission. The inability of neutralizing antibodies that block cell-free virus to interfere with spreading of certain viruses in cultures provided early evidence for cell-to-cell spread [10], [11], [12], [13], [14]. In addition, the ability of neurotropic viruses to spread along neurons or the ability of Vaccinia virus to induce actin tails that could propel viral particles to neighboring cells supported viral spread by cell-cell contact [15], [16], [17], [18], [19].

One of the best-studied viruses is the Human immunodeficiency virus (HIV) and strong support for viral spreading by cell-to-cell transmission has accumulated over the years [1], [3], [4]. HIV infection of target cells via direct cell-cell contact can be 10–1000 fold more efficient than passive dissemination of virions through the extracellular milieu [8], [9], [20], [21], [22]. HIV spreading in cell culture has also been observed to be resistant to neutralizing antibodies and to the antiviral drug tenofovir, which efficiently inhibit cell-free HIV [12], [20], [23], [24]. The current concept to explain these observations can be described by the virological synapse, a virus-induced synaptic-like contact between infected cells and uninfected target cells [23], [25], [26], [27], [28], [29], [30], [31]. The virological synapse is believed to efficiently coordinate several steps of the viral life cycle [1], [3], [4]. Tight cell-cell contacts can explain why neutralizing antibodies have limited access to cell-free virus transmitted at the cell-cell interface. Cell-cell contact sites may allow for the transmission of multiple viruses generating a high local MOI [32], [33], a phenomenon that has also been vividly documented in time-lapse videos monitoring multiple transmission events at cell-cell contact sites [23], [34], [35].

While the evidence for cell-to-cell transmission is strong and accumulating, it is not without controversy. First, in a head-to-head comparison of HIV and HTLV transmission, HIV was observed to spread mostly by a cell-free mode [36]. Second, in contrast to the higher proviral HIV content found in tissues and in co-cultures [32], [33], circulating human lymphocytes were found to carry only one provirus, which might be more consistent with infections by cell-free HIV [37]. Third, conflicting observations have been reported about the ability of neutralizing antibodies to block cell-to-cell transmission [20], [21], [38], [39]. Fourth, restriction factors such as tetherin and TRIM5 have been observed to be ineffective against infection in co-culture conditions [40], [41], yet their role as restriction factors is well established [42], [43], [44], [45], [46]. Potentially, a versatile virus like HIV could use both modes of transmission to spread, but the conditions that govern either mode are poorly understood.

**Figure 1:**
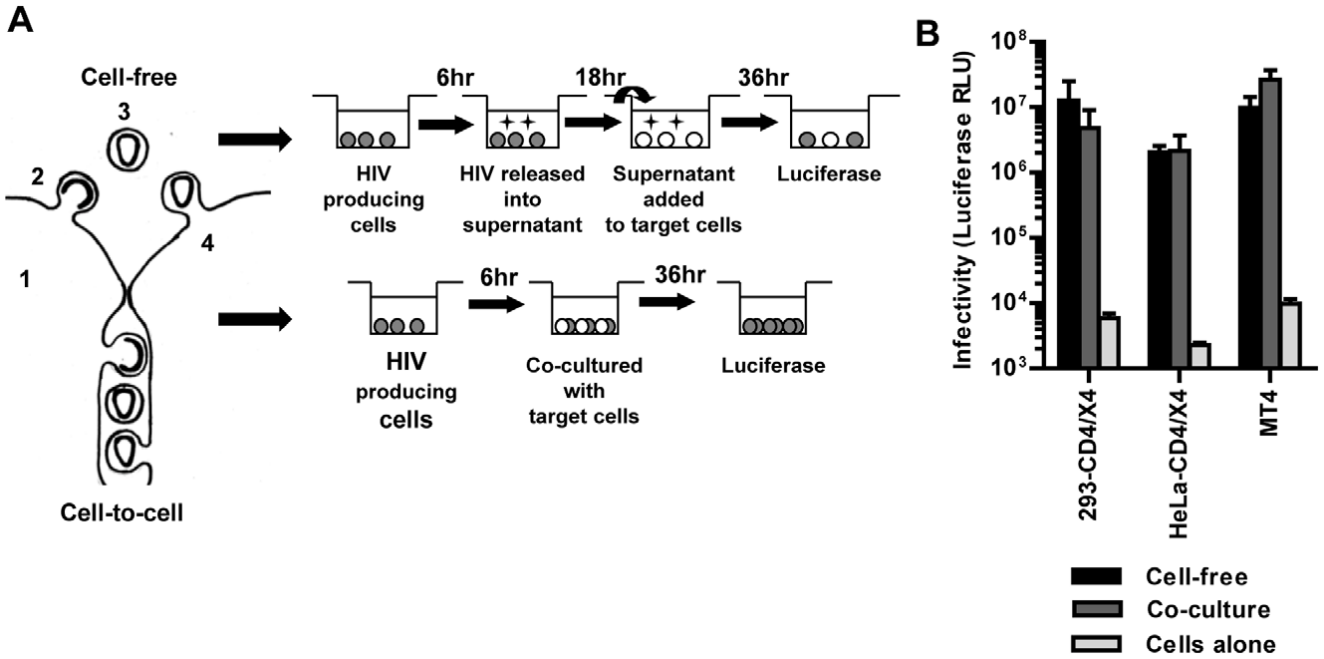
Experimental approach for comparing HIV transmission by cell-free virus or by transmission in co-cultures. (**A**) Left panel: Schematic illustration of cell-free and cell-to-cell transmission. Viruses should be able to spread by cell-free transmission if individual steps of the viral life cycle are efficient (1 = viral gene expression, 2 = virus release, 3 = stability of extracellular virus, 4 = virus entry into target cell). Right panel: Schematic illustration for the experimental comparison of HIV transmission by cell-free virus or by transmission in co-cultures using the same pool of transfected virus-producer cells. See text for details. (**B**) Transmission of HIV_NL4-3-GLuc_ by cell-free or in co-cultures were compared between highly permissive donor HEK293 and target cells (HEK293 and HeLa cells expressing CD4 and CXCR4 receptors, MT4 T cells). Infectivity was measured as relative light units of luciferase (RLU). Error bars represent the standard error of the mean from 2–8 experiments.

Given the continued controversy, we followed a systematic approach to better understand the conditions that drive virus to spread by a cell-free or a cell-to-cell mode of transmission. We reasoned that any virus should be able to efficiently spread by a cell-free mode if the following criteria are met (Figure 1A): 1) Viral gene expression should be high to promote virus assembly and release from the infected donor cell, 2) once assembled, viruses should be released efficiently into the extracellular space, 3) extracellular viruses need to be stable, and 4) viruses must bind efficiently and enter target cells. Indeed, using HIV as a model virus we demonstrate that if all the above criteria are met, this virus can efficiently spread by a cell-free mechanism. We subsequently tested if cell-to-cell transmission could overcome limitations in the cell-free path by artificially placing barriers into each of these five steps. We observed that co-culture of HIV donor cells with specific T cells can overcome each barrier imposed on the cell-free mode of virus transmission. Interestingly, the ability of co-cultures to overcome these barriers was critically dependent on the donor and target cell types. Our work places the relative contributions of cell-free versus cell-to-cell transmission of virus on a theoretical basis, which will allow other researchers to identify the underlying mechanisms of why a particular virus in a specific cell type uses either mode of transmission.

## Results

### HIV can Efficiently Spread by a Cell-free Mode if All Steps of the Replication Cycle are Efficient

To systematically place barriers into the cell-free mode of HIV transmission, we first identified a co-culture system with highly permissive cells in which HIV can spread by a cell-free mode. We choose HEK293 cells as donor cells because they support high viral gene expression and fulfilled the donor cell criteria required for efficient transmission by a cell-free mode. As target cells we tested HIV receptor/co-receptor expressing HEK293 and HeLa cells as well as transformed MT4 T cells that, in contrast to many other T cell lines, were expected to be highly permissive to cell-free HIV [36], [47], [48]. To quantitatively measure HIV cell-to-cell transmission, we transiently transfected donor cells with full-length HIV_NL4-3_ plasmid as well as an intron-regulated luciferase reporter construct [36], then co-cultured these donor cells with target cells (Figure 1A). The intron suppresses the luciferase expression in the donor cells. Luciferase expression depends on splicing of the intron from the mRNA, packaging into virions and transfer to target cells where the reporter is expressed exclusively. As such, syncytia-formation between donor and target cells does not result in luciferase expression [36]. The second generation of these constructs boosts the signal ∼1000-fold over its predecessor by using more efficiently spliced introns and by taking advantage of *Gaussia* luciferase, which is secreted from infected cells and accumulates in the culture supernatant. The luciferase signal increases linearly with increasing MOI when MT4 and Jurkat T cells are used as target cells (Figure S1A). By using this construct, we were able to quantify HIV transmission from donor cells to target cells with unprecedented sensitivity.

To measure the efficiency of cell-free and cell-to-cell transmission we had to establish experimental conditions that allow an approximate comparison of both transmission routes. This is experimentally difficult because both routes are fundamentally distinct. In the cell-free mode, HIV is released over time into the culture supernatant and the total accumulated infectivity is tested at the end. In contrast, during co-culture, viral particles can be transferred continuously from the producer cell to the target cell. Transwells containing membranes that allow the continuous passage of viruses but not cells, have been used in the past to address this problem experimentally [21]. In our experience the volume dependence of diffusion in large transwells introduces a bias towards a contact-dependent interpretation (Figure S1B–D). Therefore, we adjusted our experimental approach to allow a comparison between endpoint measurements for cell-free infection and co-cultures. This is possible because HIV is not rapidly inactivated in its cell-free form. The rate of decay for HIV was ∼10 fold over 18 h in agreement with previous reports (Figure S1E) [49], [50]. While this is a considerable rate, its consequences can be limited by short co-culture incubation times (Figure 1A, see below). Moreover, unlike vaccinia virus, which spreads faster in cultures by short-circuiting replication steps [51], the kinetics of HIV infection are largely identical under cell-free and cell-to-cell conditions (Figure S1F).

We performed co-culture experiments by transfecting HEK293 cells with pNL4-3 and the HIV-inGLuc reporter (HIV_NL4-3-GLuc_). 6 h post-transfection, we split the producer cells in half. One sample was co-cultured with target cells for 36 h before the generated luciferase was measured (Figure 1A). In parallel, the other sample was cultured for 18 h to produce viral supernatant for cell-free infections, and the viral supernatant was titered on MT4 cells by measuring luciferase 36 h post-infection to assess the released infectivity. Importantly, the signal measured at the end of the co-cultures originates from viruses that entered target cells many hours earlier because it takes time to enter cells, reverse transcribe the genome, enter the nucleus, integrate into chromosomal DNA, express the reporter gene and secrete *Gaussia* luciferase. We determined that it takes ∼18 h before the first luciferase activity can be measured in either mode of transmission (Figure S1F–G). Thus, to compare the relative infectivity of cell-free HIV produced by HEK293 producer cells with the infectivity that spreads in co-cultures, we harvested the viral culture supernatant 18 h earlier than the co-culture (Figure 1A). Applying these experimental conditions to the transmission from HEK293 producer cells to receptor/co-receptor expressing HEK293, HeLa and MT4 target T cells, we found that viral spread under both conditions was within the same order of magnitude (Figure 1B). Although our results do not exclude the contribution of cell-cell contact for the infection of these target cells because the co-culture is a mix of cell-free as well as cell-to-cell transmission, our results demonstrate that HIV can spread relatively efficiently by a cell-free mode under the combination of highly permissive donor and target cells.

### Co-culture can Partially Overcome Low Viral Gene Expression

**Figure 2:**
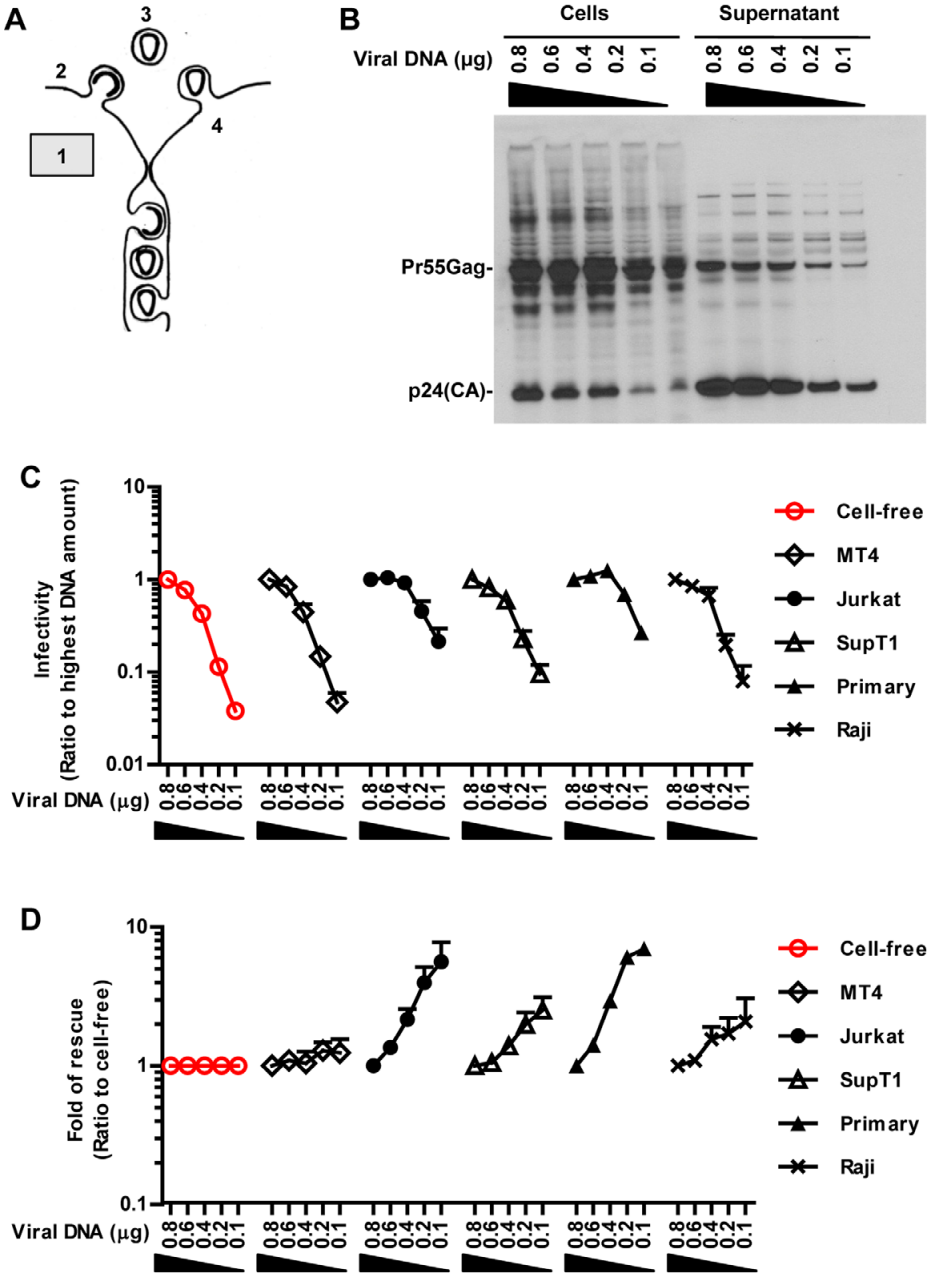
Co-culture can overcome low viral gene expression. (**A**) A barrier was placed into the cell-free path of HIV by lowering viral gene expression. (**B**) HEK293 producer cells transfected in 12-well plates with decreasing amounts of viral plasmid DNA (µg) were incubated for 30 h and cell lysates and viral supernatant were analyzed by Western blot using α-p24 antibodies. (**C**) Relative HIV_NL4-3-GLuc_ infectivity released by HEK293 producer cells (red) or transmitted from producer cells to indicated target cell types in co-cultures. Infectivity measured for the highest amount of transfected DNA was set to 1. (**D**) Fold of rescue of co-culture over cell-free from (C). Fold-rescue was determined by calculating the ratio of the infectivity in co-cultures over the infectivity of cell-free virus at the corresponding plasmid dose. Error bars represent the standard error of the mean from 3 experiments.

To next understand the underlying steps that influence the transmission of HIV by cell-cell contact, we placed barriers that affect the infectivity released into the viral supernatant and asked how the cell-cell spread of infectivity was affected by this perturbation. We had reasoned that high viral gene expression is needed to support efficient virus assembly and release (Figure 2A). To hinder this first step, we progressively lowered viral gene expression in HEK293 cells by transfecting decreasing amounts of pNL4-3 into producer cells while keeping the total DNA constant. This resulted in a decline in the total production of HIV from HEK293 cells and a corresponding decline in infectivity of the culture supernatant when tested on MT4 cells (Figure 2B, C). MT4 cells were used as reporter cell lines to monitor changes in the infectivity of cell-free virus released into the viral supernatant due to their high susceptibility to HIV infection (Figure 1B). Co-culture of HEK293 cells with MT4 cells did not change the observed decline in HIV infectivity (Figure 2C). In contrast, exploring co-cultures with various target cells, we observed that viral spreading in co-cultures with Jurkat T cells and primary T cells was significantly more resistant to the lowering of viral gene expression (Figure 2C). This relative resistance to lowering of viral gene expression was best illustrated as fold-rescue by normalizing the declining infectivity in co-cultures to the declining infectivity of cell-free HIV (Figure 2D).

### Co-culture Can Overcome the Tethering of Viruses to the Producer Cell

**Figure 3:**
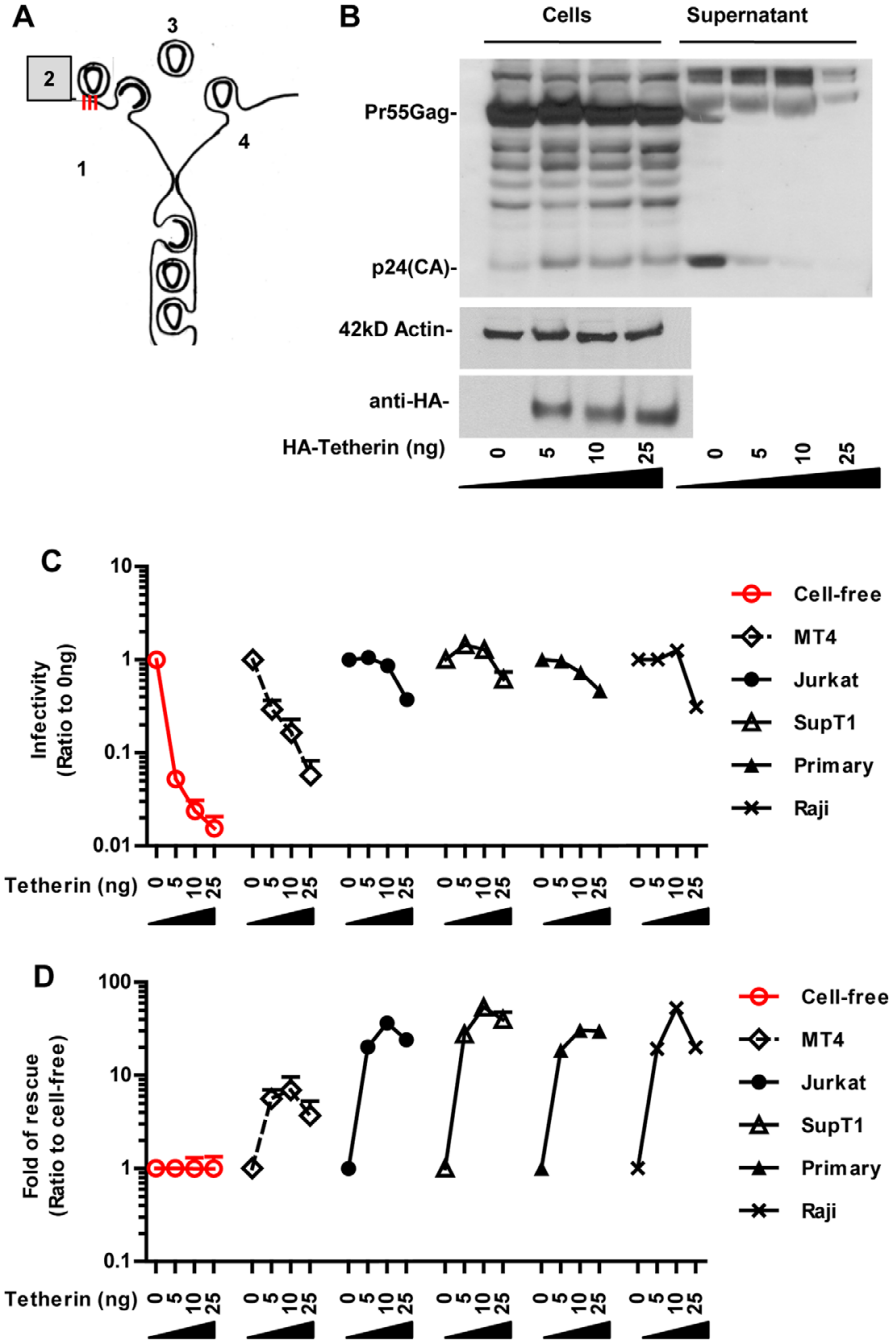
The reduction in release of cell-free HIV_Δvpu_ by tetherin is overcome in co-cultures. (**A**) A barrier was placed into the cell-free path of HIV at the step of virus release by expressing tetherin. (**B**) HEK293 cells producing HIV_LAI-Δvpu_ and increasing amounts of tetherin (ng) were analyzed for viral gene expression in cells and virus release into the culture supernatant by Western blotting using the α-p24 antibody. The expression of HA-tagged tetherin was confirmed using α-HA antibodies. (**C**) Relative HIV_LAI-Δvpu-GLuc_ infectivity released by HEK293 producer cells expressing increasing amounts of tetherin (red) or transmitted from producer cells to indicated target cell types in co-cultures. The ratio of infectivity to non-tetherin expressing cells was calculated. (**D**) Fold of rescue of co-culture over cell-free for the data shown in (C). Fold-rescue was determined by calculating the ratio of the infectivity in co-cultures over the infectivity of cell-free virus at the corresponding tetherin plasmid dose. Error bars represent the standard error of the mean from 3 experiments. The effects of tetherin expression on wild-type HIV_LAI_ are presented in Figure S2.

Efficient spreading by a cell-free mode requires that viruses are released into the extracellular space (Figure 3A). To interfere with the release of HIV from the surface of producer cells, we expressed increasing amounts of “tetherin”, which restricts the release of HIV lacking the accessory protein Vpu [46], [52], [53]. As expected, expression of increasing amounts of tetherin in HEK293 producer cells placed a barrier into the release of cell-free HIV lacking Vpu (Figure 3B and C), but not wild-type virus (Figure S2). Co-culture with MT4 cells relieved the block of HIV lacking Vpu mildly (Figure 3C). In contrast, co-cultures with other T cells, including Jurkat and primary T cells relieved the block from tetherin expression strongly (Figure 3C). The rescue of infectivity was clearly seen when we normalized the infectivity measured in co-culture to that of cell-free infectivity (Figure 3D).

### Co-culture can Overcome the Sensitivity of Cell-free Virus to Neutralizing Antibodies

**Figure 4:**
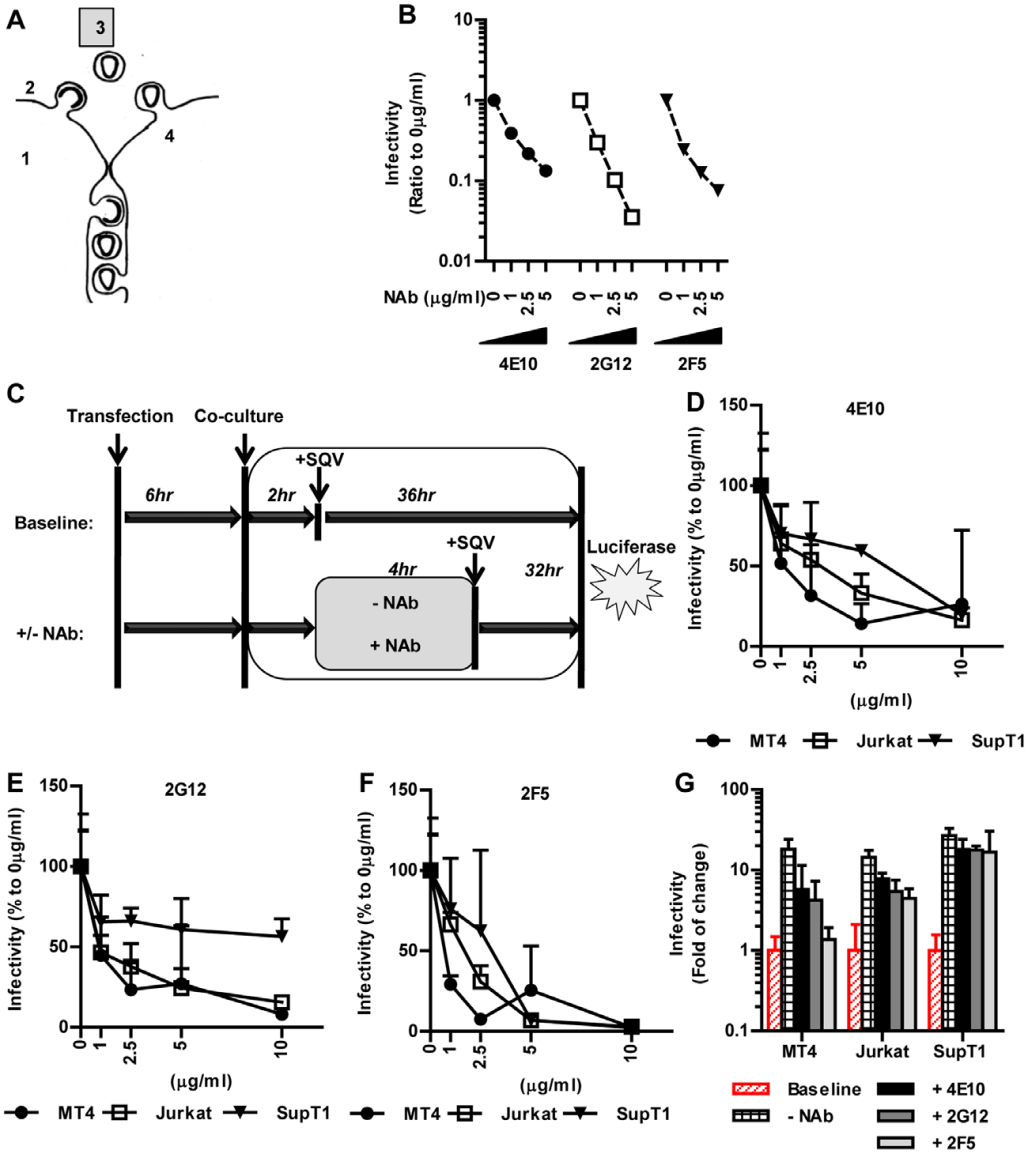
Sensitivity of HIV transmission by cell-free virus or by transmission in co-cultures to neutralizing antibodies. (**A**) A barrier was placed into the cell-free path of HIV at the extracellular step using neutralizing antibodies. (**B**) The infectivity of cell-free HIV_NL4-3-GLuc_ on MT4 cells was measured in the absence and presence of increasing amounts of neutralizing antibodies (NAb) 4E10, 2G12, and 2F5 (µg/ml). Infectivity was normalized to 1 using mock-treated control. (**C**) Scheme depicts the experimental approach to test the sensitivity of HIV spreading in co-cultures to neutralizing antibodies. At 6 h post-transfection, donor and target cells were co-cultured and cell-cell contacts were allowed to form for 2 h. Transmission events occurring during these 2 h were measured by terminating transmission with saquinavir (SQV) and allowing all previous infection events to proceed for 36 h and result in luciferase expression. These base level infections were set to 1. In parallel, the ability of neutralizing antibodies (NAb) to interfere with the spreading of infection to indicated cell types during a window of 4 h was determined. At the end of the 4 h incubation transmissions were terminated using SQV and the produced luciferase was determined 32 h later. (**D–F**) Dose response for indicated neutralizing antibodies 4E10, 2G12, 2F5 in co-cultures of HEK293 HIV-producer cells with the indicated target cell lines as described in (C). (**G**) The data point at 2.5 µg/ml from figures D–F. Infectivity is normalized to the baseline infection levels measured during the first 2 h of co-culture. Error bars represent the standard error of the mean from 3 experiments.

After the virus is released into the extracellular space, it has to remain stable long enough to reach target cells by random diffusion. The inactivation rates for cell-free HIV were not fast enough to prevent a cell-free mode of spreading under our experimental conditions (Figure 1B and S1E). To make the cell-free extracellular virus vulnerable at this step, we utilized the neutralizing antibodies 2G12, 4E10 and 2F5 [54], [55], which all inhibit cell-free HIV infection in a dose-dependent manner (Figure 4A, B). We then asked if co-culture could overcome this barrier. Cell-cell contact between HIV infected cells and uninfected target cells is mediated by Env-CD4 interactions [20], [27], [28]. Consequently, addition of anti-Env or anti-CD4 antibodies prior or at the time of initiation of co-culture prevented the spreading of HIV (data not shown). To specifically ask if the infectivity of cell-free virus or if cell-to-cell transmission are differentially susceptible to neutralizing antibodies, we first allowed cell-cell contacts to form over a period of 2 h and then tested if neutralizing antibodies could prevent the further spreading of infectivity over a 4 h window (Figure 4C). This was achieved experimentally by terminating the generation of any new infectious viruses using the protease inhibitor saquinavir while allowing already transmitted viruses to complete the infection and to produce luciferase in target cells (Figure 4C). This approach specifically addresses whether neutralizing antibodies can disrupt already established cell-cell contacts. In the absence of any neutralizing antibodies, the infectivity increased over 10 fold during this 4 h window (Figure 4G). The extent by which the addition of increasing amounts of neutralizing antibodies inhibited HIV cell-to-cell transmission over this 4 h window was dependent on the cell-type and the antibody (Figure 4D–F). Co-cultures between HEK293 donor and MT4 target cells remained most sensitive to all three neutralizing antibodies (Figure 4D–F). In contrast, co-cultures with Jurkat and SupT1 T cells revealed increasing resistance of HIV transmission to the neutralizing antibodies (Figure 4D–F), especially at lower concentrations (1 and 2.5 µg/ml (Figure 4G)). Moreover, antibody 2F5 was more effective at interfering with HIV cell-to-cell transmission than 4E10 and 2G12. Thus, cell-to-cell transmission to Jurkat and SupT1 cells remained more resistant to neutralizing antibodies suggesting that these cells can form stronger synapses as compared to sensitive MT4 cells.

### Co-culture can Overcome an Entry Barrier into Poorly Susceptible T Cells

**Figure 5:**
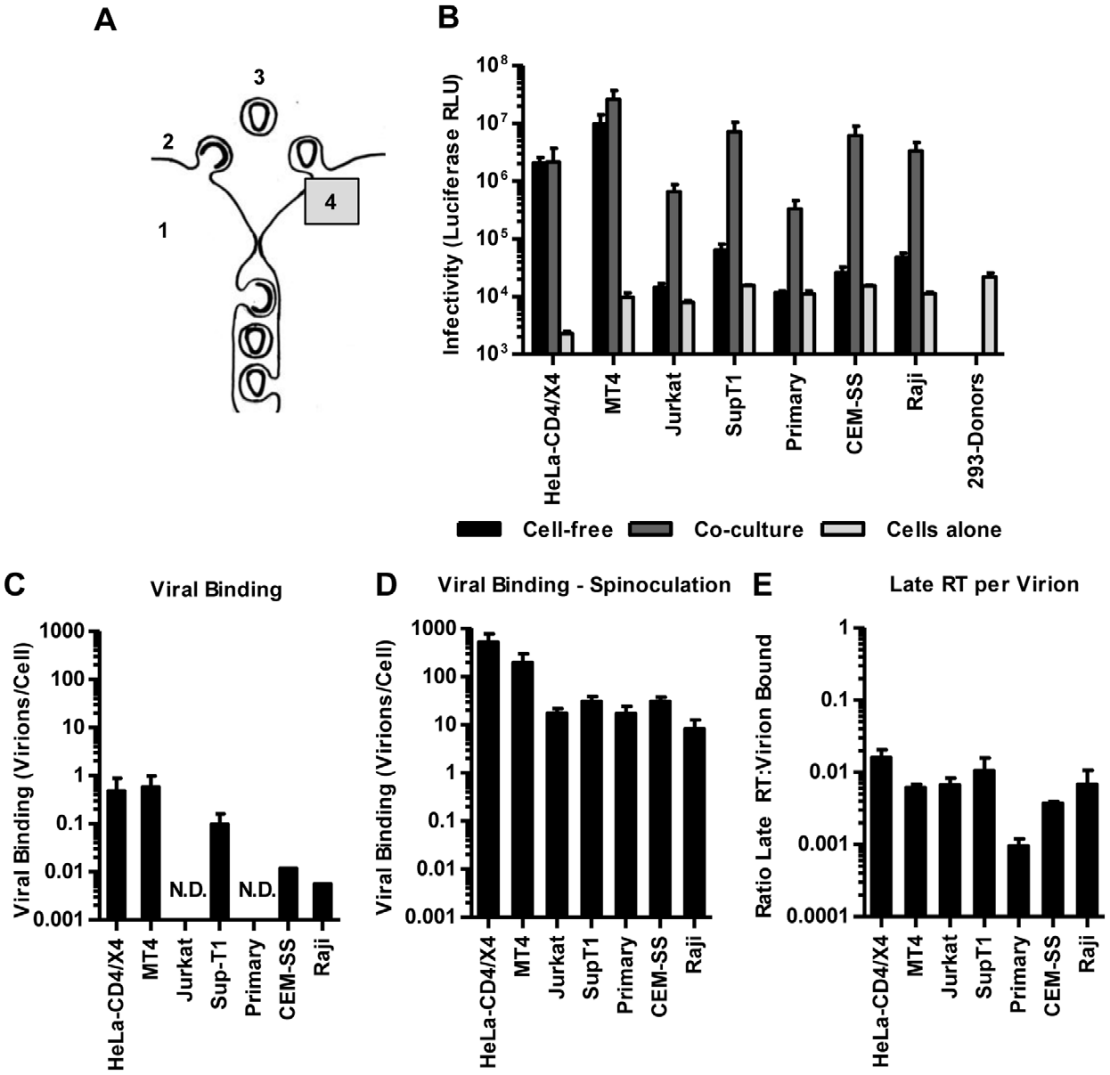
Co-culture can overcome an entry barrier into poorly susceptible T cells. (**A**) The existence of potential entry barriers in the cell-free path of HIV transmission was explored by varying target cells. (**B**) An experiment as in (Figure 1B) was performed to compare cell-free HIV_NL4-3-GLuc_ infection and spreading infections in co-cultures of HEK293 donor cells with indicated target cells. For comparison, the data for permissive HeLa and MT4 from Figure 1B are shown to the left. Error bars represent the standard error of the mean from 7–8 experiments. (**C**) The binding capability of cell-free HIV_NL4-3-GLuc_ on indicated cell types was tested at 37°C for 2 h (∼9 ng/ml of p24). The cells were then washed to remove unbound particles. Cells were then lysed and analyzed by α-p24-ELISA. “ND” for “non-detectable” by ELISA in >600,000 infected cells. Error bars represent the standard error of the mean from 2 experiments. (**D**) Relative virus binding was measured as in (C) for indicated cell types following spinoculation with concentrated HIV_NL4-3-GLuc_ virus (∼600 ng/ml). (**E**) Late reverse transcription was measured following incubation at 37°C for 36 h. Data shown in D–E were determined in parallel within one experiment and 3 experiments were combined. Error bars represent the standard error of the mean.

Following the HIV life cycle, cell-free virus needs to then efficiently bind and enter the target cell to spread the infection (Figure 5A). We systematically compared the differential susceptibility of various target cells to cell-free infection as well as cell-to-cell transmission in co-cultures (Figure 5B). As discussed above (Figure 1B), HIV can spread efficiently by cell free mode from highly permissive cell types such as HEK293 to HIV receptor-expressing HeLa and MT4 T cells (Figure 5B). In striking contrast, all other cell types tested were poorly susceptible to cell-free HIV despite the fact that equal amounts of the same viral supernatant efficiently infected HeLa and MT4 cells (Figure 5B). Importantly, direct co-culture of these target cells with donor cells overcame the entry barrier reaching infection levels similar to that observed in highly permissive cells (Figure 5B). This includes activated primary human CD4^+^ T cells, which became susceptible to infection only under co-culture conditions. These data indicate that several target T cells exhibit a relatively strong barrier to cell-free HIV that is likely an important cause for the observed contact-dependent mode of HIV transmission.

The relative resistance of target cells to cell-free infection is likely caused by less efficient viral binding. To test this we incubated target cells with 9 ng/ml of p24 of cell-free HIV_NL4-3-GLuc_ at 5×10^5^ cells/ml and incubated at 37°C for 2 h. This inoculation matches the average inoculum used during the above cell-free infection conditions (Figure 5B). Subsequently cells were washed to remove unbound virus, lysed and the bound p24 measured using an HIV p24-specific ELISA assay (Figure 5C). Virus bound most efficiently to the highly permissive HeLa and MT4 cells. In contrast, binding to Jurkat and primary T cells was too poor for detection indicating that inefficient binding contributes to the relatively low susceptibility of both cell types to cell-free HIV. Receptor availability does not fully account for this observation since we did not observe a strong correlation between the susceptibility of the different cell types and the level of receptor/co-receptor expression (Figure S3A, B). A stronger correlation with CD4 levels was only observed within isogenic clones derived from Jurkat E6.1 cells that express different receptor/coreceptor levels (Figure S3C, D).

To be able to measure the efficiency of post-binding events we delivered ∼600 ng/ml (p24) of concentrated HIV_NL4-3-GLuc_ onto cells at 2–6×10^6^ cells/ml by spinoculation. This method increases viral binding without significantly affecting the susceptibility of the cells [48], [56], [57]. ELISA for bound p24 indicated that HeLa and MT4 cells were still more efficient in capturing HIV, but spinoculation allowed us to overcome the binding barrier of cell-free HIV on Jurkat and primary T cells (Figure 5D). Following spinoculation, cells were then incubated for another 36 h and measured the number of late reverse transcription products per cell (Figure 5E). We found that reverse transcription was relatively less efficient in primary cells compared to the other cell types. This suggests that both binding and post-binding events are less efficient in primary cells. Altogether, our findings suggest that cell-to-cell transmission overcomes viral binding and post-binding barriers in target cells.

### The Actin Cytoskeleton can Represent an Additional Barrier to Cell-free HIV

**Figure 6:**
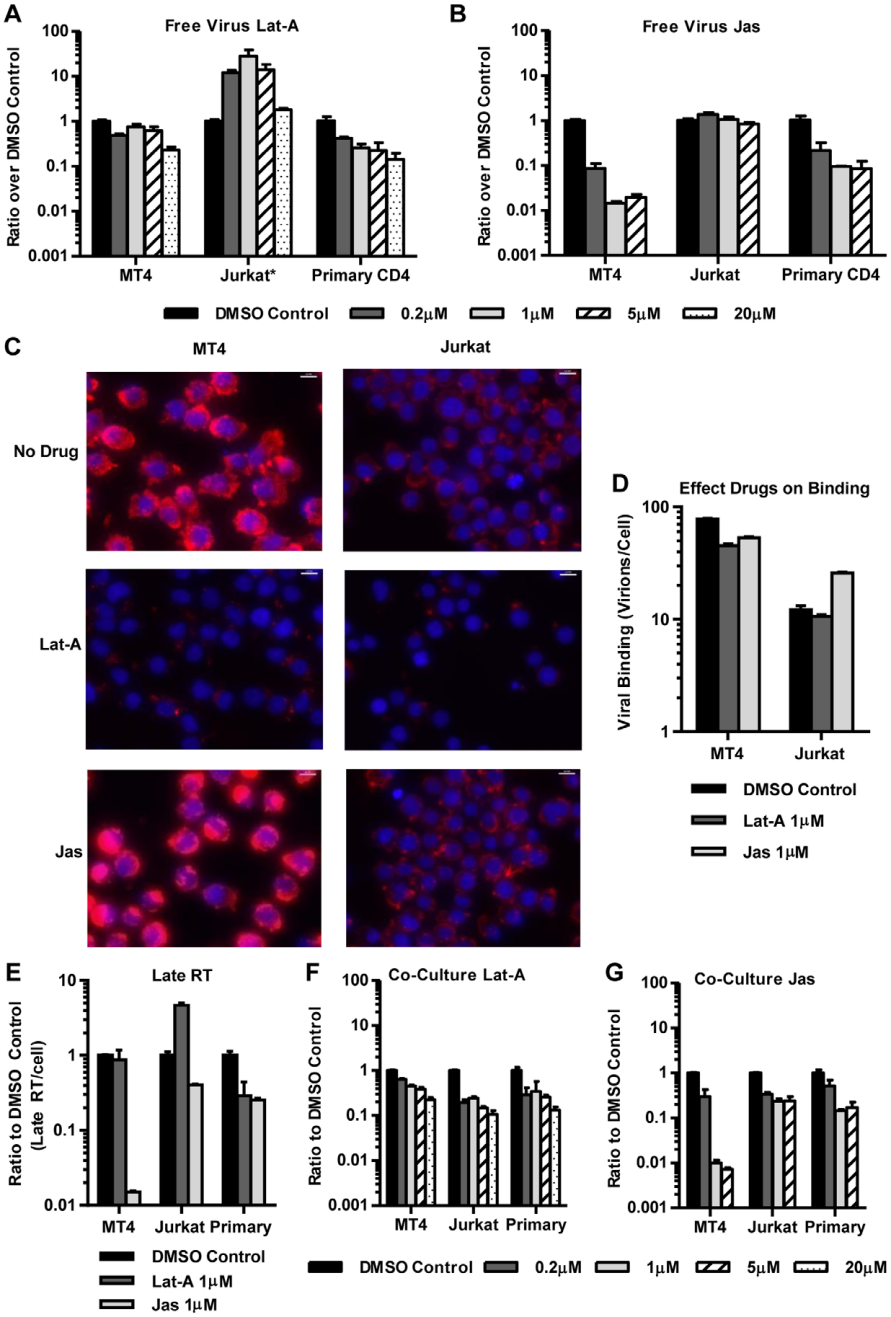
The actin cytoskeleton of Jurkat cells presents a barrier to cell-free HIV infection. (**A, B**) MT4, Jurkat and primary CD4 T cells were inoculated with concentrated cell-free HIV_NL4-3-GLuc_ by spinoculation and incubated at 37°C in the presence or absence of increasing concentrations of latrunculin-A (Lat-A) or japlakinolide (Jas)(µM). Luciferase activity was measured 36 h post-inoculation. Data were normalized to DMSO control. Error bars represent the standard error of the mean from 2 experiments. (**C**) Phalloidin staining of untreated, Lat-A-, and Jas-treated cells. Cells were exposed to Lat-A (1 µM for MT4 and 0.5 µM for Jurkat) or Jas (0.5 µM for MT-4 and 0.0625 µM for Jurkat) for 1 h at 37°C. Note that phalloidin competes with Jas for binding to polymerized actin and further dilution of drug was required to observe actin staining in Jurkat cells [76]. Size bars correspond to 10****µm. (**D**) Viral binding was measured by α-p24-ELISA after spinoculating cells in the presence or absence of 1 µM Lat-A or Jas. Error bars represent the standard deviation from 3 measurements. (**E**) Late reverse transcription (RT) was measured by Q-PCR from cells treated with 1 µM of Lat-A or Jas. Error bars represent the standard deviation of 3 late RT measurements. (**F, G**) A co-culture experiment as in Figure 5B was performed in the presence of increasing concentrations of Lat-A or Jas (µM). Error bars represent the standard error of the mean from 2 experiments.

The filamentous actin cytoskeleton (F-actin) has been described to represent a barrier for cell-free HIV in quiescent T cells [58], [59]. To test if F-actin poses a similar barrier to cell-free HIV in other commonly used T cell types, we added increasing amounts of the actin-depolymerizing agent latrunculin A (Lat-A), or the actin-stabilizing drug jasplakinolide (Jas) to cells and tested their susceptibility to concentrated cell-free HIV. The observed phenotypes varied strongly among cell types. In Jurkat cells, the dissolution of the actin cytoskeleton using Lat-A enhanced their permissiveness to cell-free HIV_NL4-3-GLuc_ up to ∼30-fold, but Jas exhibited little effect (Figure 6A, B). In contrast, in highly permissive MT4 cells, Lat-A had no effect, but treatment with the actin-stabilizing drug Jas made these cells ∼100-fold more resistant to cell-free HIV (Figure 6A, B). Parallel phalloidin staining for F-actin documented the effectiveness of the drug treatments on both cells types (Figure 6C, data not shown). Both, Lat-A and Jas inhibited HIV infection of primary CD4 T cells (Figure 6A, B). This indicates that the actin arrangements in activated primary cells are not a pre-existing barrier to cell-free HIV infection. However, both depolymerization (Lat-A) and stabilization (Jas) of actin in primary cells introduces a barrier to cell-free infection. The remaining cell types used in this study exhibited phenotypes similar to primary cells but not as striking as in Jurkat or MT4 cells (data not shown).

We tested which entry step was affected by drug treatment and observed only a mild ∼2-fold enhancing effect on HIV binding to Jurkat cells for Jas and a mild inhibitory effect on HIV binding to MT4 cells for both Lat-A and Jas (Figure 6D). More pronounced effects that largely explained the overall phenotype were observed at the level of reverse transcription (Figure 6E). These experiments suggest that the nature of the cortical actin cytoskeleton contributes to the differential susceptibility to cell-free HIV. In fact, depolymerizing the actin cytoskeleton made Jurkat cells more MT4-like and stabilizing actin made MT4 cells more Jurkat-like. Unlike observations in quiescent T cells, where HIV entry is influenced by the activation of cofilin [58], cofilin is activated in Jurkat cells and does not contribute to this phenotype (data not shown).

The actin cytoskeleton plays an important role in the formation of the virological synapse [23], [27], [60], [61]. To confirm the importance of actin during cell-cell transmission under our experimental conditions we tested the effects of the actin inhibitors Lat-A and Jas on HIV cell-to-cell transmission. We found that spreading of virus in co-cultures with Jurkat and primary T cells remained actin-dependent irrespective of the whether actin was de-polymerized or stabilized (Figure 6F, G). Thus, the enhancement of HIV entry into Jurkat T cells seen for cell-free HIV, is not observed in co-cultures suggesting that the cortical actin cytoskeleton is reorganized. In contrast, the phenotype observed in co-cultures with MT4 cells largely phenocopied the results with cell-free HIV.

Altogether, our findings suggest that the organization of F-actin can pose a significant barrier to the entry of cell-free HIV in some T cells. This is especially clear in Jurkat T cells, which display a similar phenotype as reported for quiescent T cells [58], [59]. This actin barrier to cell-free infection is likely overcome by active actin remodeling mediated by cell-cell contact [20], [27], [60].

### Donor Cell Induced Contact Dependence

**Figure 7:**
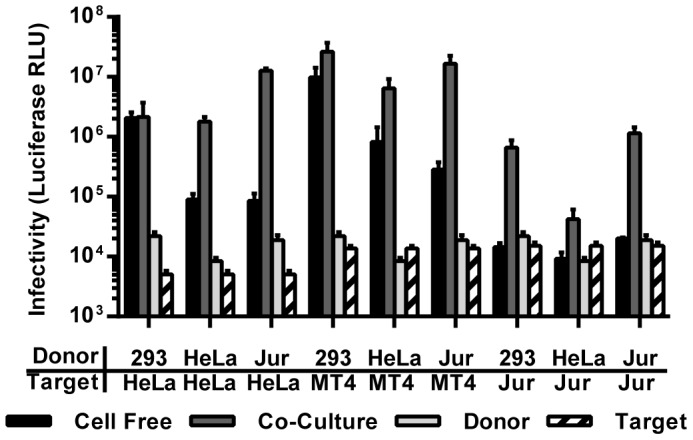
The relative contribution of cell-free to co-culture mediated transmission is affected by the donor cell type. Different donor cells (HEK293, HeLa, Jurkat) were co-cultured with HeLa cells expressing CD4/CXCR4, MT4 cells and Jurkat cells. The efficiency of virus transmission in the cell-free mode and in the co-culture-dependent mode was compared as described in Figure 1A. Data for HEK293 donor cells are as Figure 5B. Error bars represent the standard error of the mean from 2 experiments.

By varying target cells we found that entry barriers for cell-free HIV in specific T cells imposed a contact-dependent mode of transmission on HIV. However, varying the donor cells can likely also affect contact dependence. Based on the effects of the barriers placed onto highly permissive HEK293 donor cells, we would expect that specific donor cells with less capacity to support the assembly and release of cell-free HIV would also induce a more contact dependent mode of spreading. To test this hypothesis we directly compared HEK293 with HeLa and Jurkat T cells as donors in co-culture experiments. As expected, HeLa and Jurkat T cells produced and released ∼10- and ∼40-fold less cell-free HIV, respectively (Figure 7). However, co-cultures of Jurkat donor cells with HeLa, MT4 and Jurkat target cells resulted in ∼60–100-fold more efficient spreading. Transfer from Jurkat donor cells to Jurkat target cells was at least as efficient as using HEK293 donor cells. Switching from HEK293 donor cells to HeLa and Jurkat donor cells even introduced a significant contact-dependence on the spreading of HIV to HeLa and MT4 cells, which are highly permissive to cell-free HIV (Figure 7). The only exception was the transmission from HeLa to Jurkat cells, which was not very efficient. While the luciferase signal was too low when primary cells were used in our experimental system, our data provide a proof of concept for scenarios of donor cell-induced contact dependence.

### The Concentration of Viral Gene Products at Sites of Cell-cell Contact may Contribute to the Efficiency of Cell-to-cell Transmission

**Figure 8:**
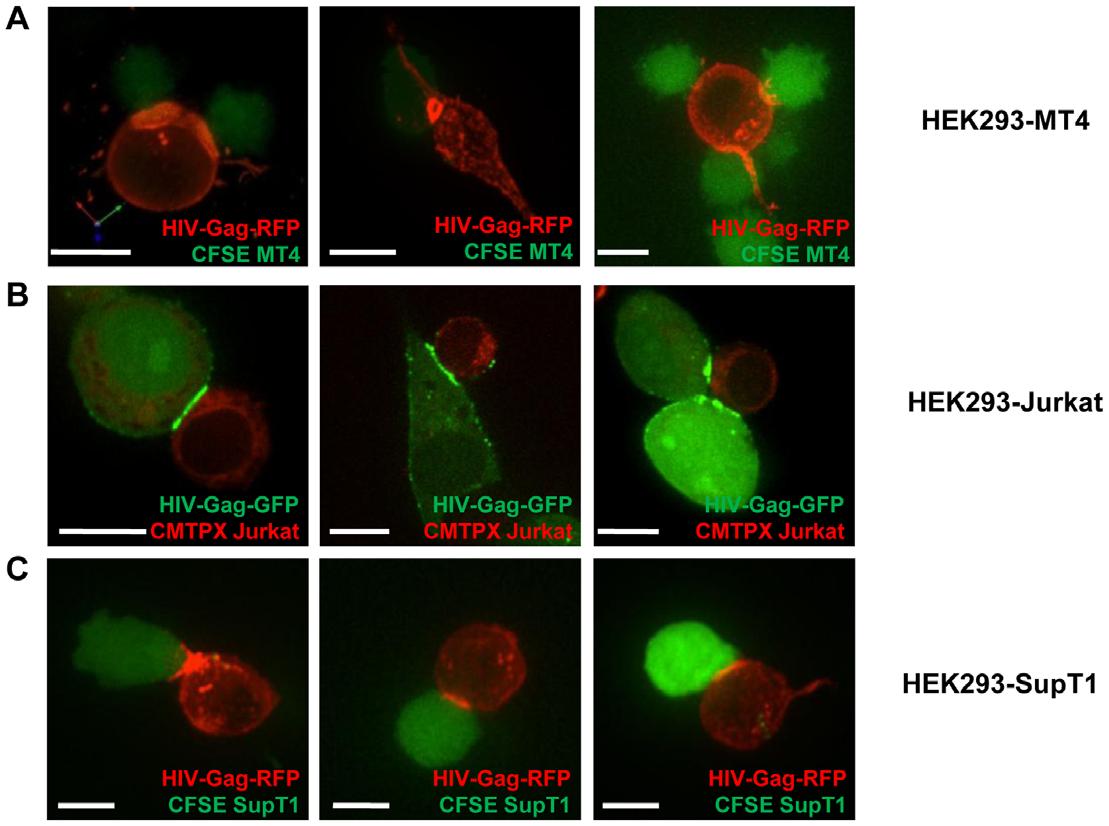
Concentration of HIV-Gag to sites of cell-cell contacts. (**A–C**) HEK293 cells were co-transfected with HIV_NL4-3_ and fluorescently tagged HIV_NL4-3-GFP_ (green) or HIV_NL4-3-RFP_ (red) and co-cultured with the dye-labeled target T cells MT4 cells (CFSE, green)(A), Jurkat cells (CMTPX, red))(B), and SupT1 cells (CFSE, green)(C) and imaged by confocal fluorescence microscopy. Size bars represent 10 µm.

The observed ability of co-cultures to efficiently overcome barriers to the cell-free mode of HIV transmission is likely explained by the formation of virological synapses [25], [26], [27]. Virological synapses form between an infected cell and a receptor-expressing target cell and can efficiently coordinate several steps of the viral replication cycle [1], [2], [3], [4]. Assembly and budding of viruses, rather than being distributed across the plasma membrane, can be locally concentrated either by sequestration of surface particles or by *de novo* assembly at sites of cell-cell contacts [23], [35], [62]. We expressed full-length HIV_NL4-3_ carrying Gag-GFP or -RFP (HIV_NL4-3-GFP_ and HIV_NL4-3-RFP_ respectively) fusion proteins at the position of Pol in HEK293 producer cells and co-cultured them with dye labeled MT4, Jurkat and SupT1 T cells. We observed the concentration of HIV Gag at sites of cell-cell contact in all co-cultures (Figure 8A–C). The structures included button- and ring-like structures as well as poly-synapses as previously reported (Figure 8A) [23], [28]. These experiments suggest that a local concentration of viral factors at sites of cell-cell contact can contribute to the ability of cell-to-cell transmission to overcome low viral gene expression in the donor cells.

### Co-culture Promotes Spreading of HIV to More Cells and They Carry an Increased Proviral Content

**Figure 9:**
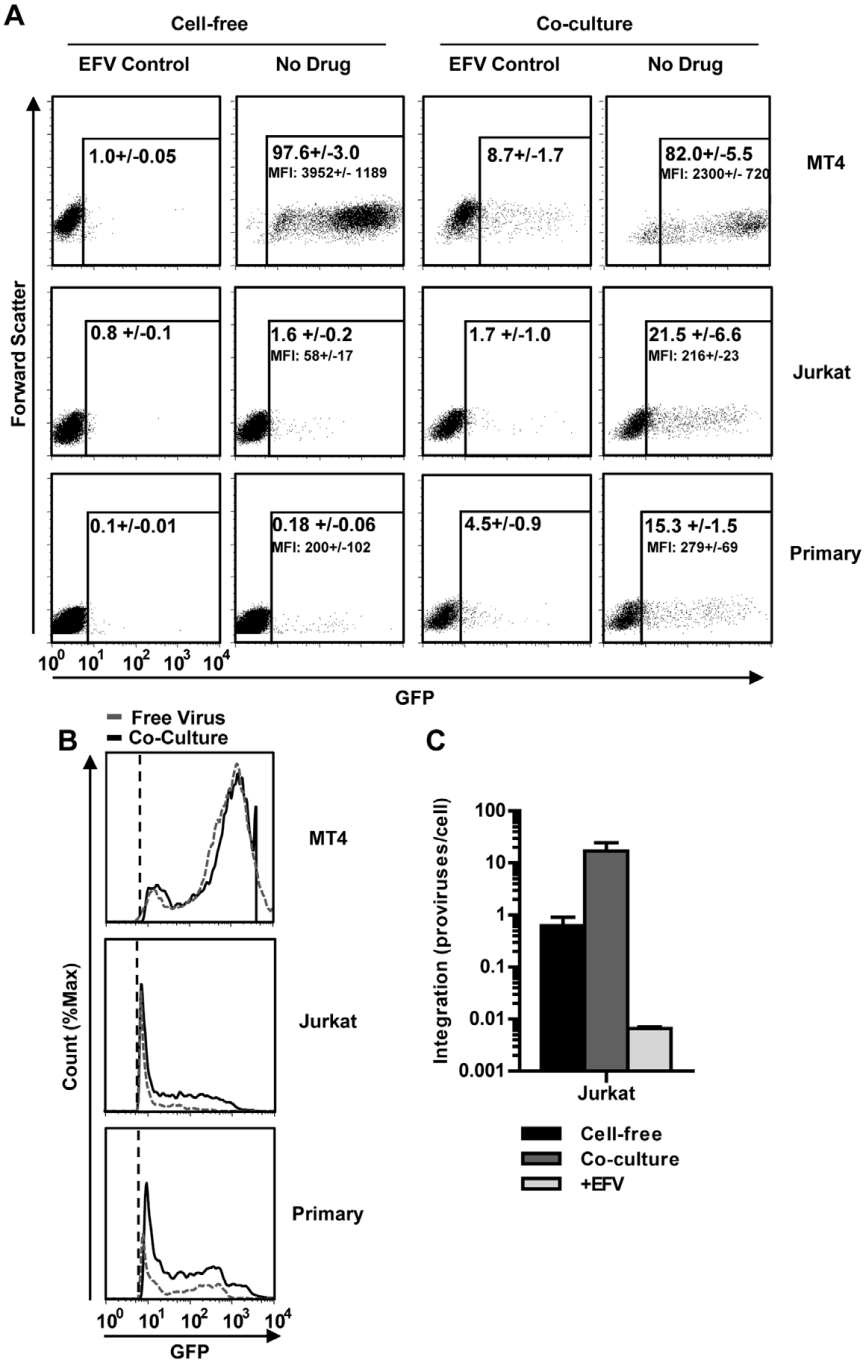
Co-culturing cells leads to a larger proportion of infected target cells and a higher proviral content. (**A**) Infection by cell-free or by co-cultures with MT4, Jurkat or primary CD4+ T cells were repeated as in Figure 5B using HEK293 donor cells producing HIV_IRES-GFP_. Target cells were identified based on their expression of CD2 (MT4 cells) or CD3 (Jurkat and primary CD4 T cells). GFP fluorescence was gated based on the background fluorescence from a corresponding sample treated with 1 µM of efavirenz (EFV). Numbers represent the average percent of GFP-positive cells +/− the standard deviation of 2 (efavirenz control) or 6 (no drug) inoculations. MFI values correspond to the average mean fluorescence +/− the standard deviation of 2 (efavirenz control) or 6 (no drug) inoculations. (**B**) Comparison of the GFP fluorescence intensity of cells infected by cell-free virus or by co-culture from the gated population in panel (A). Histograms represent the combination of 3 measurements. Fluorescence was normalized to the fluorescence from the corresponding efavirenz-controls to account for fluorescence shifts due to sample variability. The grey dashed line represents cell-free infection and solid black line represent co-culture infection. The black vertical dashed line represents the limit of the gates shown in (A). (**C**) An experiment as in (A) was repeated with wild-type HIV_NL4-3_ and target cells were stained with CFSE. CFSE-positive cells expressing HIV Gag above the efavirenz-treated control (see Figure S5) were sorted and the number of HIV integration events was analyzed by *Alu*-PCR. Control samples were treated with 1 µM efavirenz (+EFV). Error bars represent the standard deviation of 3 measurements of integration.

In order to further understand why cell-to-cell transmission leads to efficient viral spreading, a single cell-based assay was needed. Measuring luciferase released into the supernatant, while highly sensitive, is a population based assay and cannot distinguish between three distinct scenarios: 1) that high luciferase levels are the result of more cells expressing similar levels of luciferase, 2) few highly infected cells are expressing high levels of luciferase, or 3) a combination of both. To address this question, we applied an HIV *nef*-IRES-GFP construct (HIV_IRES-GFP_) [63] and measured the number of dye-labeled target cells expressing GFP after co-culture with donor HEK293 cells or infection with cell-free virus. Efavirenz-treated cultures served as controls. These experiments revealed that highly susceptible MT4 cells were efficiently infected by cell-free HIV produced by HEK293 cells (Figure 9A). In contrast, while cell-free HIV infected only about ∼1% of Jurkat cells and 0.2% of primary CD4 T cells, co-cultures increased the number of infected cells by ∼10–100 fold, respectively (Figure 9A). These experiments demonstrate that the high efficiency of HIV cell-to-cell transmission is, at least in part, caused by efficient spreading of HIV to more cells.

We noted that the intensity of GFP (MFI) in target cells increased in co-cultures (Figure 9B). Because GFP is under the control of the HIV LTR in HIV_IRES-GFP_, an increase in intensity should correlate with an increase in proviral copies per cell. Unlike HIV Gag, GFP is not released from infected cells and continues to accumulate over time (Figure S4A). Thus, this observation would suggest that co-cultures may not just lead to the infection of more cells, but that these cells are also more highly infected. To test this possibility, we asked if GFP intensity correlates with the proviral copy number. Towards this end, we sorted Jurkat cells infected with HIV_IRES-GFP_ based on GFP fluorescence intensity and determined the provirus copy number using *Alu*-PCR (Figure S4B). We found that there is a direct correlation between proviral copy number and GFP fluorescence intensity in Jurkat cells (Figure S4C). This allowed us to determine how the proviral copy number increased in co-cultures with Jurkat cells (Figure 9B). We estimated that the proviral copy numbers reached ∼18 proviruses/cell when Jurkats were co-cultured with donor HEK293, while cell-free virus infection was below the limit of detection (<9 proviruses/cell – Figure S4C).

Since estimating proviral copy number based on GFP fluorescence intensity has a limited dynamic range, we confirmed these initial observations using an alternative approach. We performed cell-free and co-culture infections with wild-type HIV_NL4-3_ and FACS-sorted CFSE dye-labeled target T cells based on HIV Gag expression (Figure S5). Because co-cultures result in the transfer of HIV Gag material from donor cells to target cells in the absence of infection [20], we again used efavirenz treated co-cultures as controls (Figure S5). We performed *Alu*-PCR on sorted Gag-expressing cells to measure the number of integration events. *Alu*-PCR is a very sensitive and specific method for measuring HIV integration. This method can detect 1 integration event in 10,000 cells and has a dynamic range of 5–6 orders of magnitude [57]. These results revealed proviral numbers of ∼1 provirus/cell for cell-free HIV infection and ∼16 proviruses/cell in Jurkat cells infected in co-cultures (Figure 9C). Thus, these experiments demonstrate that co-culture conditions increase both the number of infected target cells and the number of proviral copies per target cell.

## Discussion

### A Model for the Relative Contribution of Cell-free and Cell-to-cell Transmission to Viral Spreading

**Figure 10:**
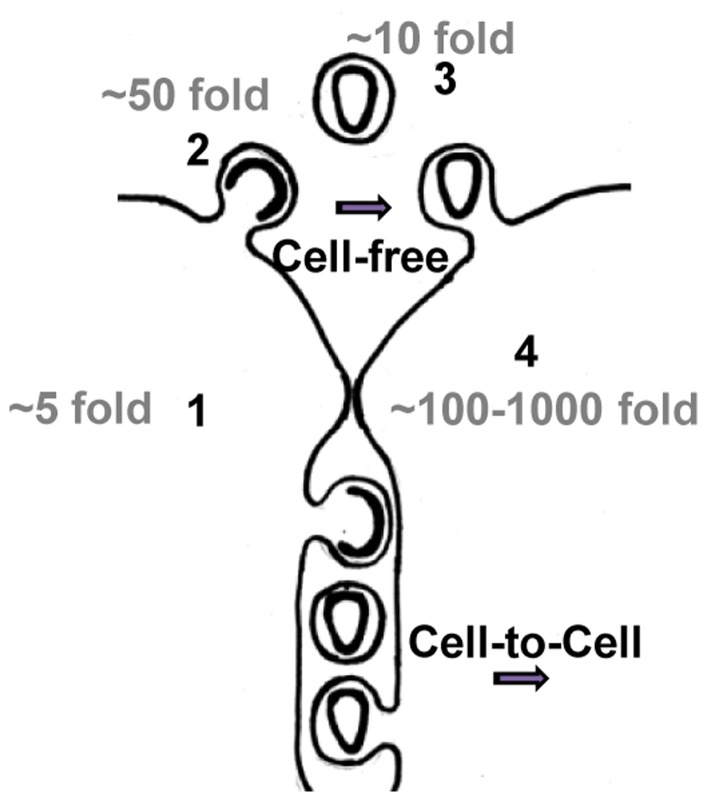
Size of barriers experimentally introduced into the cell-free path of HIV. By interfering with the cell-free path of HIV, these barriers tilt virus transmission towards contact-dependent modes. See text for details.

We have used HIV as a model virus to understand the conditions that results in viral spread by cell-free or cell contact-dependent mode. We found that if all steps of the viral replication cycle are efficient, HIV can spread by a cell-free mode. However, if any of these steps is impaired, co-cultures of HIV donor cells with specific target cells allows efficient viral spreading. This rescue of HIV infectivity is likely mediated by the transfer of particles across a virological synapse. Experimentally, we introduced barriers that compromised the cell-free path of HIV by as low as 5-fold by lowering viral gene expression in donor cells, ∼50 fold by expressing tetherin in donor cells or as high as 2–3 orders of magnitude by using poorly susceptible target T cells (Figure 10). In all cases, co-cultures of HIV donor cells with specific cell types were able to overcome these limitations in the cell-free pathway. Our simple experimental approach illustrates how the effects of several barriers in the cell-free path can accumulate and result in strong contact dependence. In light of these numbers, experimentally observed contact dependences above 100-fold for HIV and up to 10,000-fold for the Human T cell lymphotropic virus (HTLV-1) appear plausible [7], [8], [9], [20], [36]. Our work on the relative contributions of cell-free versus cell-to-cell transmission to viral spreading will help identify the underlying mechanism of why a particular virus in a specific cell type uses either mode of transmission.

### Donor and Target Cell Induced Contact Dependence

We demonstrate that barriers in the cell-free path can be strong enough to force virus transmission into a contact-dependent mode. Depending on whether these barriers are located on the donor or target cell, we would describe them as either donor or target cell-induced contact-dependences. HTLV-1 transmission with its proposed roles for Tax, cytoskeletal polarization, O-glycosylation and biofilm biogenesis in the donor cell suggests the existence of a pronounced donor cell-induced contact-dependence [64], [65], [66]. In contrast, many T cells including primary human CD4^+^ T cells are poorly permissive to cell-free HIV, which effectively blocks the cell-free mode of infection and promotes a target cell-induced contact-dependent mode of spreading. By varying the HIV donor cell, we were able to present a proof of concept that donor cell types that produce less cell-free virus, as observed in Jurkat T cells, can similarly impose a contact-dependent mode of spreading on HIV. Thus, Jurkat cells exhibited both, a pronounced donor as well as target cell-induced contact dependence and promoted the efficient spread of HIV in the absence of any detectable cell-free virus transmission.

### The Ability of Cell-to-cell Transmission to Overcome Barriers in the Cell-free Path is Influenced by the Cell-type

We explored the contribution of each step in the viral life cycle to affect the mode of transmission. We performed new experiments as well as reviewed and quantified previously reported observations in our model system. We demonstrate how low viral gene expression in the producer cell impair the cell-free mode of HIV dissemination, yet still permit robust HIV transmission in co-cultures. We reproduced the previously reported inability of tetherin [40] and neutralizing antibodies [12], [20], [23] to interfere with HIV cell-to-cell transmission, but also demonstrate that these observations depend on the cell type and the antibody used. We show that strong virological synapses formed with T cells become increasingly resistant to the glycan-recognizing antibody 2G12 as well as the gp41-targeting antibody 4E10 in contrast to previous reports that suggested that cell-to-cell transmission is only resistant to antibodies recognizing the CD4-binding site [39]. Finally, we show that the poor susceptibility of T cells to cell-free HIV promotes cell-to-cell transmission. The demonstration of the ability of cell-to-cell transmission to overcome these various barriers also recalls previous reports that the restricting ability of TRIM5 and the enhancing effects of Nef on HIV infectivity can similarly be overcome in co-cultures [41], [67]. Importantly, by varying cell types in all our experiments, we document that the ability of HIV cell-to-cell transmission to overcome barriers in cell-free transmission depends on the cell type. This offers a possible explanation for conflicting reports by various groups [12], [20], [21], [23], [36], [38], [40], [68], [69]. Specifically, co-cultures with Jurkat, SupT1 and primary T cells can efficiently overcome barriers imposed on the donor cell, yet co-cultures with MT4 cells largely fail (Figures 2, 3, 4). Moreover, in contrast to Jurkat, SupT1 and primary cells, HIV transmission to MT4 cells remains more sensitive to neutralizing antibodies. Collectively, these experiments suggest that HIV spreads from permissive donor cells to MT4 cells largely by a cell-free mode of transmission. However, if combined with donor cells such as HeLa cells and Jurkat cells, HIV transmission to MT4 cells can become contact-dependent due to a donor cell-induced effect (Figure 7). These data illustrate again that an observed contact-dependence in a particular combination of cell types can be contributed from either the donor, the target cells or both.

### Measuring the Strength of Virological Synapses as a Criterion for Contact-dependence

MT4 cells are highly permissive to cell-free HIV, but this alone cannot explain their inability to overcome strong barriers imposed on the donor cell. We hypothesized that this is because they cannot establish strong virological synapses. Visually, all cell types were able to concentrate Gag at the cell-cell interface, indicating that imaging may not be a sufficiently accurate indicator for cell-to-cell transmission. Rather, we propose that our functional assays are better indicators for cell-to-cell transmission since we can assess which barriers to cell-free dissemination can be overcome by cell-cell contact. Consistent with this observation, co-cultures with Jurkat and SupT1 cells were increasingly resistant to neutralizing antibodies and Jurkat cells and primary T cells carried a high proviral content providing proof for high local MOI as previously observed [33]. We believe that these functional assays, the ability to overcome barriers in the donor cell (low viral gene expression and tetherin expression), the resistance to neutralizing antibodies, and the number of proviruses in the target cell provides several criteria to determine if a particular virus in a particular cell type combination either preferentially spreads by a cell-free or by a cell-to-cell mode of transmission.

## Materials and Methods

### Cells

Peripheral blood mononuclear cells were purified from whole blood (New York Blood Center) with the Ficoll-Paque Plus gradient (GE Healthcare Life Sciences). Following this purification step, CD4 T cells were purified using the EasySep Human CD4+ T Cell Enrichment Kit (StemCell Technologies) and were stimulated with PHA (10 µg/ml) (Sigma-Aldrich), IL-2 (100 U/ml), IL-7 (100 ng/ml), and IL-15 (100 ng/ml) for 72 h (cytokines from Miltenyi Biotec). The cell lines Jurkat E6.1 (ATCC), MT4 (NIH AIDS Research and Reagent Program), Sup-T1 (NIH AIDS Research and Reagent Program), Raji-CD4 (NIH AIDS Research and Reagent Program), CEM-SS (NIH AIDS Research and Reagent Program) and HEK293 (ATCC) were maintained in RPMI (Gibco), supplemented with 100 U/ml penicillin/streptomycin (Gibco), 2 mM of L-glutamine, and 10% FBS (Gibco). The HeLa-derived cell line TZMbl (a gift from Vineet KewalRamani, NCI Frederick) was maintained in DMEM (Gibco) supplemented with 100 U/ml of penicillin/streptomycin (Gibco), 2 mM of L-glutamine, and 10% FBS (Gibco). Jurkat E6.1 cells acquired from ATCC were heterogeneous on their CD4 expression levels and were sorted into a homogeneous population of CD4+ cells (CD4++) using an Aria (BD Biosciences) cell sorter (Figure S6).

### Plasmids

To obtain HEK293 CD4/X4 cells, HEK293 cells were transfected with plasmids encoding human CD4 and human CXCR4 using Fugene 6 (Roche). Cells were stained for CD4 and CXCR4 and analyzed by flow cytometry using a FACS-Calibur (BD Biosciences). The cells were incubated at 37°C for 24 h prior to conducting co-culture experiments. The plasmid encoding the intron-regulated HIV-based *Gaussia* luciferase [70] pUCHR-inGLuc (HIV-inGLuc) was generated in the Derse/Heidecker lab as previously described for the firefly luciferase-containing construct [36]. Transcription of GLuc is antisense relative to transcription of the viral genomic RNA and is interrupted by a γ-globin intron inserted in sense orientation relative to the genomic RNA. The plasmid encoding the HIV molecular clone NL4-3 and plasmid encoding HIV-IRES-GFP (pBR43IeG-nef+) were obtained from the AIDS Research and Reagents Program. The constructs encoding the HIV clones LAI and LAI-Δ*vpu* were a gift from Michael Emerman, Fred Hutchinson Cancer Research Center, Seattle [71]. HIV-Gag-GFP was described previously [72]. HIV-Gag-RFP was a gift from Akira Ono, University of Michigan, Ann Arbor [73]. Plasmid encoding the vesicular stomatitis virus G-glycoprotein (VSV-G) was obtained from Michael Marks, University of Pennsylvania. Tetherin was transiently expressed using pCR3.1/HA-based plasmids, which were donated by Paul Bieniasz, Aaron Diamond AIDS Research Center, New York [52].

### Reagents

Latrunculin A and jasplakinolide were purchased from Enzo Life Sciences. The following reagents were obtained from the NIH AIDS Research and Reagent Program: zidovudine, efavirenz, saquinavir, raltegravir, HIV-neutralizing antibodies (2F5, 4E10, 2G12), and anti-CXCR4 antibody (clone 12G5). Anti-CD4 antibody (clone RPA-T4) was purchased from eBiosciences. Antibody against HIV Gag (clone KC57) was purchased from Beckman Coulter. Luciferase activity was measured using the BioLux *Gaussia* Luciferase Assay Kit (New England Biolabs).

### Co-culture Experiments

Co-culture experiments were performed using HEK293 cells transiently transfected with the plasmids encoding full length HIV (molecular clone NL4-3) and HIV-inGLuc (at a ratio of 6∶1) using Fugene 6 Transfection Reagent (Roche) or XtremeGene9 (Roche). In the cases where HeLa cells were used as donor cells, Fugene HD was used for transient transfection (Promega). The kinetics of spreading HIV infectivity of cell-free versus cell-associated HIV were determined to establish the following protocol (Figure S1E-G Figure 1A): 6 h post-transfection, the transfected cells were split into halves. One half of the producer cells (5×10^5^/ml) were co-cultured with target cells (10^6^/ml) for 36 h at 37°C at a donor-to-target ratio of 1∶2. Infectivity was assessed by luciferase activity. The viral supernatant produced from the other half of HEK293 producer cells cultured alone was harvested 18 h later, to correct the kinetics of infection of target cells in the co-culture (Figure 1A). Viral supernatant was added to target cells and incubated for 36 h at 37°C followed by measuring luciferase activity. In transwell assays, the 12 mm Transwell® with 3.0 µm pore polycarbonate membrane insert (Corning #3402) was used.

### Concentrated Cell-free Virus Infections

Viral supernatants from HEK293 cells transfected with pNL4-3 and HIV-inGLuc (6∶1) were concentrated by ultracentrifugation over a cushion of 20% sucrose solution in PBS or by using the Lenti-X Concentrator (Clontech). Cells were resuspended in concentrated viral supernatant at 2–6×10^6^ cells/ml to obtain a monolayer of cells. Cells were then spinoculated at 1200 × g for 2 h at room temperature in flat bottom wells as similarly described [56]. Following spinoculation, fresh medium was added to the cells and incubated at 37°C for 36 h. For single round infections, virus was washed after inoculation with PBS +10% FBS and resuspended in fresh medium containing 1.25 µM of saquinavir.

### Measuring Viral Binding

Viral binding was measured from inoculated cells as similarly described [57]. Briefly, cells were washed with PBS +10% FBS after inoculation to remove unbound particles. Cells were then lysed in PBS +0.5% Triton-X and assayed for HIV p24 by ELISA (Advanced BioScience Laboratories, Inc.). Virions bounds per cell were calculated assuming 15800 particles per pg of p24 [74].

### Measuring HIV DNA Intermediates

HIV late reverse transcription was measured by Q-PCR as similarly described [75] using BioRad’s iCycler 3. The PCR conditions for the late reverse transcription assay are 25 µl of DNA sample in 10 mM Tris-HCl pH8 with 25 µl of master mixture at 2× concentration: 100 mM KCl, 40 mM Tris-HCl (pH 8), 11 mM MgCl_2_, 0.6 mM of each dATP, dCTP, dGTP and dTTP, 0.52 µM of forward (5′-GCCTCAATAAAGCTTGCCTTGAGTG-3′) and reverse (5′-CAGCAAGCCGAGTCCTGCG-3′) primers, 0.4 µM of TaqMan probe (5′-FAM-CCAGAGTCACACAACAGACG-TAMRA-3′) (Integrated DNA Technologies) and 2.5 U of Platinum Taq Polymerase (Life Technologies). Conducted the following PCR program: hot-start at 95°C for 2 min, followed by 50 cycles of denaturation for 15 sec at 95°C, annealing for 15 sec at 60°C, plate read and extension for 1 min at 72°C. The number of HIV copies was determined using pNL4-3 as a standard. HIV molecules were normalized to copies per cell by albumin Q-PCR following the same PCR conditions as late reverse transcription with the following primers and probe: 5′-GCTGTCATCTCTTGTGGGCTGT-3′ (forward), 5′-AAACTCATGGGAGCTGCTGGTT-3′ (reverse) and 5′-FAM-CCTGTCATGCCCACACAAATCTCTCC-TAMRA-3′ (TaqMan probe) (Integrated DNA Technologies). HIV integration was measured as previously described [57] using 2.5 U of Platinum Taq (Life Technologies).

### Measuring HIV Integration from Sorted Samples

Cells infected by co-culture or cell-free virus (following same co-culture protocol described above) were fixed with BD Cytofix/Cytoperm (BD Biosciences) solution overnight at 4°C. Cells were washed with 1× BD Perm/Wash (BD Biosciences) solution and incubated with PE-labeled anti-HIV Gag antibody (KC57-RD1 Beckman Coulter) in BD Perm/Wash solution for 30 min at 4°C. Washed cells with BD Perm/Wash solution and resuspended in FACS Buffer (PBS +0.5% BSA +2 mM EDTA) at 10^7^ cells/ml. HIV-positive cells were sorted using a Sony iCyt-Reflection cell sorter. In co-culture experiments, target cells were stained with CFSE (Molecular Probes) to distinguish target from donor cells. Cells treated with 1 µM efavirenz (AIDS Reagents Program) were used as negative control. Following the sort, cells were spun, resuspended in 200 µl of PBS +200 µl of Buffer AL (Qiagen) +20 µl of Proteinase K (Qiagen) and incubated at 60°C for 24 h to remove paraformaldehyde. DNA was purified using the DNeasy Blood and Tissue Kit (Qiagen).

### Staining Filamentous Actin

Cells were incubated at 37°C for 1 hr in the presence of different concentrations of latrunculin-A and jasplakinolide. Cells were then washed with PBS and fixed with 100 µl of BD Cytofix/Cytoperm solution at room temperature for 20 min. After fixation, the cells were washed with BD Perm/Wash solution and resuspended in 100 µl of BD Perm/Wash solution containing 0.2 µM of phalloidin (Molecular Probes) and 0.5 µg/ml of Hoechst 33342 for 30 min at room temperature. Cells were washed with BD Perm/Wash and resuspended in PBS. Cells were imaged by confocal microscopy on LabTek-II chamber slides (Nunc) using a Nikon Eclipse TE 2000-U microscope at 60 × magnification. Images were analyzed using OpenLab software (Improvision). Images shown represent the dose of latrunculin-A that inhibited actin staining. Phalloidin competes with jasplakinolide for actin binding [76]. The images shown represent the dose of jasplakinolide with the strongest actin staining.

### Co-culture Experiment with Jurkat Cells as Donors

A population of Jurkat cells carrying HIV-inGLuc was developed to serve as donor cells. To establish this population, Jurkat E6.1 cells with low or no expression of CD4 were sorted to prevent re-infection with HIV. The HIV-inGLuc construct carrying blasticidin resistance was transfected into cells using TransIT-Jurkat transfection reagent (MirusBio). Cells were grown under blasticidin (5 µg/ml) (Invivogen) selection for >2 weeks. This population of cells was infected with concentrated HIV_NL4-3_ (VSV-G) by spinoculation and incubated for 24 h post-inoculation in the presence of 6 ng/ml of phorbol 12-myristate 13-acetate (PMA) and 0.25 µM of ionomycin. Infected cells were then washed and co-cultured with target cells at 1∶1 ratio for 36 h. Virus produced from donor cells over 24 h was used for cell-free inoculations of target cells.

### Spinning-disc Confocal Imaging

3D images made from spinning-disc microscope was performed at 37°C by using a 60 × oil objective (numerical aperture, 1.4) of a Nikon TE2000 inverted wide-field microscope equipped with Piezo drive or by using an Improvision spinning disc confocal microscope equipped with a Nikon TE2000 base as previously described [35]. Cells were plated on 35 mm imaging dishes (MatTek, Ashland, MA) coated with poly-L-lysine and 0.2 mg/ml fibronectin in RPMI with 10% FBS plus penicillin-streptomycin-glutamine as previously described. HIV-Gag-GFP or HIV-Gag-RFP were used to transfect HEK293 cells and target cells were stained with CMTPX 5 µM (Molecular Probes) or CFSE 0.5 µM (Molecular Probes). Co-culture of producer cell (3×10^5^) and target cells (4×10^5^) from 9–12 h post-transfection and were fixed with 4% paraformaldehyde before imaging.

## Supporting Information

Figure S1 **Experimental approach to compare HIV transmission by cell-free virus or by transmission in co-cultures. (A)** Luciferase activity generated by HIV_GLuc_ correlates linearly with the proviral content. Concentrated HIV_NL4-3-GLuc_ was titered on MT4 and Jurkat T cells, cells were incubated for 36 h at 37°C in the presence of saquinavir to restrict infection to a single cycle, and HIV integration was measured by *Alu*-PCR. Cells treated with efavirenz (EFV) were used as negative controls. **(B, C)** Comparison of HIV_NL4-3-GLuc_ spreading between HEK293 donor cells and MT4 target cells in co-culture or transwell settings and controls. “CC” is the co-culture of HEK293 donor cells with MT4 target cells; “CC transwell” is the separation of co-culture using transwells where target cells were at the bottom and donor cells were on the top of the transwell; “FV transwell” is adding the virus containing supernatant (collected as is shown in Figure 1) into the transwell; parallel, “FV” is directly adding the virus containing supernatant onto the targets that were seeded on the bottom of the well. Arrow indicates the direction of the virus infectivity. Star indicates the place where the viruses are likely most concentrated. **(D)** Volume dependence of HIV_NL4-3-GLuc_ infection of MT4 cells. A constant amount of HIV_NL4-3-GLuc_ was added to a constant number of MT4 target cells and the final volume of the culture was adjusted to the indicated values. The luciferase activity was measured 36 h post-inoculation. **(E)** Stability of cell-free HIV. The infectivity of cell-free HIV_NL4-3-GLuc_ on MT4 was measured following incubation of the indicated time at 37°C in the absence or presence of HEK293 cells. Infectivity of HIV_NL4-3-GLuc_ at time point zero was set to one. Error bars represent the standard error of the mean from 2 experiments. **(F, G)** Kinetics of cell-free HIV infection and spread of infectivity in co-cultures to MT4 (F) and Jurkat T cells (G). The infectivity of cell-free HIV_NL4-3-GLuc_ (red) or transmitted to indicated target cells (green) in co-cultures was measured at indicated time points after initiation of infection. Error bars represent the standard deviation of 3 independent replicates.
(PDF)

Figure S2 **Control experiment for** **Figure 3B, C** **for HEK293 cells expressing tetherin and wild-type HIV.** **(A)** A Western blot analysis as in Figure 3B for HEK293 cells expressing wild-type HIV_LAI_ that does not lack Vpu and increasing amounts of tetherin (ng). **(B)** Relative HIV_NL4-3-GLuc_ infectivity released by HEK293 producer cells expressing increasing amounts of tetherin (red) or transmitted from producer cells to indicated target cell types in co-cultures. The infectivity was normalized to non-tetherin expressing cells. Error bars represent the standard error of the mean from 3 experiments.
(PDF)

Figure S3 **Susceptibility to HIV correlates weakly with CD4 expression among various cell types except in an isogenic background of Jurkat clones.** **(A, B)** Indicated cell types were infected by spinoculation with concentrated cell-free HIV_NL4-3-GLuc_ and luciferase activity was measured 36 h post-inoculation. Luciferase activity was correlated with CD4 and CXCR4 expression levels. R^2^ and p-values from a linear regression test are shown. **(C, D)** Individual Jurkat clones derived from Jurkat E6.1 displaying various levels of CD4 expression were spinoculated with concentrated HIV_NL4-3-GLuc_ or co-cultured with HEK293 donor cells producing HIV_NL4-3-GLuc_. The resulting luciferase activity was measured 36 h post-inoculation. Luciferase activity was correlated with CD4 and CXCR4 expression levels. R^2^ and p-values from a linear regression test are shown.
(PDF)

Figure S4 **Experimental details for correlating GFP expression levels with the numbers of HIV proviruses shown in** **Figure 9B** **.** **(A)** Comparison of HIV Gag fluorescence with GFP intensity. MT4 cells were infected with cell-free HIV_IRES-GFP_. HIV Gag (α-Gag-PE) and GFP expression (GFP) were measured 36 h post-infection. Mean fluorescence intensity of HIV Gag-PE was calculated for GFP^lo^ and GFP^hi^ populations. Figure illustrates that higher infection levels per cell are better detected from GFP fluorescence intensity than from HIV Gag-PE fluorescence intensity. **(B)** Jurkat cells were inoculated with concentrated HIV_IRES-GFP_ (VSV-G) and incubated at 37°C for 24 h. Cells were then sorted into separate populations based on GFP mean fluorescence. Sort purity is indicated in the gates of each sorted population. HIV integration was measured from the sorted populations by *Alu*-PCR. The level of proviruses/cell is indicated above each population dot plot. **(C)** The number of proviruses/cell was correlated with the GFP mean fluorescence by linear regression. The limit of detection is at ∼9 proviruses/cell based on a sample treated with 1 µM efavirenz (EFV).
(PDF)

Figure S5 **Experimental details for the determination of proviral numbers in cell-free infection or in co-cultures presented in** **Figure 9C** **.** CFSE and HIV-Gag double positive Jurkat cells were sorted from cell-free infections or co-cultures. Sorting gates were set based on a 1 µM efavirenz (EFV)-treated control. “After sort” displays the purity of the sorted cell fraction. Note that percent infection shown under “Before Sort” column may not represent actual percentage of infected cells due to interfering staining coming from bound viral particles. The number of HIV integration events of these sorted cells was analyzed by *Alu*-PCR (see Figure 9C).
(PDF)

Figure S6 **Generation of Jurkat E6.1 cells with high CD4 expression.** Jurkat E6.1 cells introduced from ATTC displayed a larger portion of cells lacking CD4 expression. Cells were sorted for CD4 expression to generate a Jurkat E6.1 population that expressed CD4 more homogeneously (CD4++). All experiments in this study were conducted with this polyclonal Jurkat CD4++ population.
(PDF)
