# Supplementary figures and images for: Cell-to-Cell Transmission Can Overcome Multiple Donor and Target Cell Barriers Imposed on Cell-Free HIV
Source: PLoS One. 2013 Jan 7;8(1):e53138. doi: 10.1371/journal.pone.0053138 (PMC3538641; doi:10.1371/journal.pone.0053138)

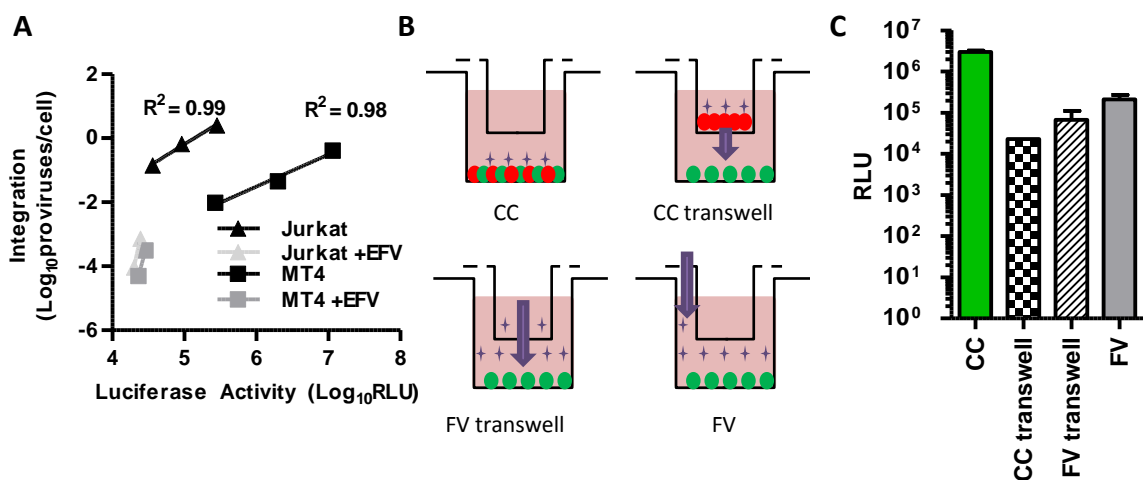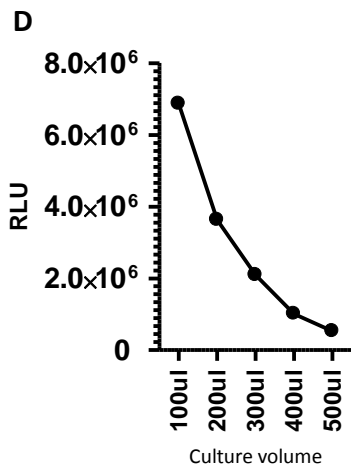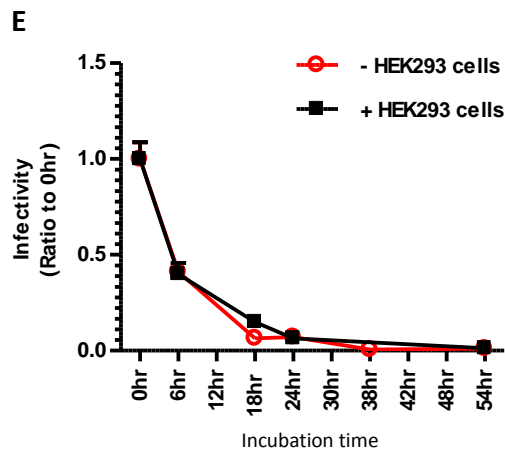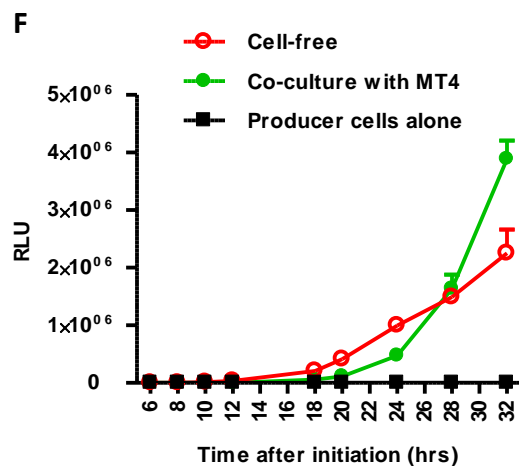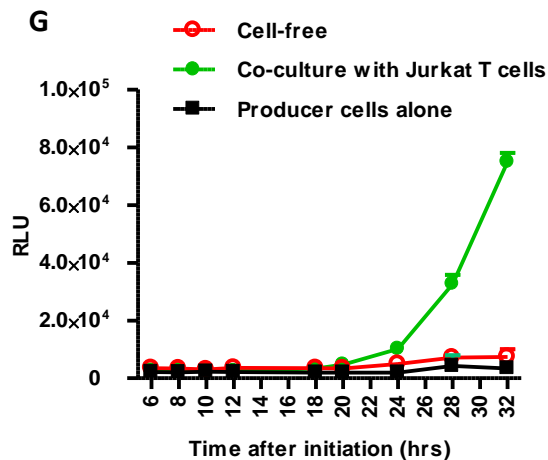

Supplement: Figure S1 — Experimental approach to compare HIV transmission by cell-free virus or by transmission in co-cultures. (A) Luciferase activity generated by HIVGLuc correlates linearly with the proviral content. Concentrated HIVNL4-3-GLuc was titered on MT4 and Jurkat T cells, cells were incubated for 36 h at 37°C in the presence of saquinavir to restrict infection to a single cycle, and HIV integration was measured by Alu-PCR. Cells treated with efavirenz (EFV) were used as negative controls. (B, C) Comparison of HIVNL4-3-GLuc spreading between HEK293 donor cells and MT4 target cells in co-culture or transwell settings and controls. “CC” is the co-culture of HEK293 donor cells with MT4 target cells; “CC transwell” is the separation of co-culture using transwells where target cells were at the bottom and donor cells were on the top of the transwell; “FV transwell” is adding the virus containing supernatant (collected as is shown in Figure 1) into the transwell; parallel, “FV” is directly adding the virus containing supernatant onto the targets that were seeded on the bottom of the well. Arrow indicates the direction of the virus infectivity. Star indicates the place where the viruses are likely most concentrated. (D) Volume dependence of HIVNL4-3-GLuc infection of MT4 cells. A constant amount of HIVNL4-3-GLuc was added to a constant number of MT4 target cells and the final volume of the culture was adjusted to the indicated values. The luciferase activity was measured 36 h post-inoculation. (E) Stability of cell-free HIV. The infectivity of cell-free HIVNL4-3-GLuc on MT4 was measured following incubation of the indicated time at 37°C in the absence or presence of HEK293 cells. Infectivity of HIVNL4-3-GLuc at time point zero was set to one. Error bars represent the standard error of the mean from 2 experiments. (F, G) Kinetics of cell-free HIV infection and spread of infectivity in co-cultures to MT4 (F) and Jurkat T cells (G). The infectivity of cell-free HIVNL4-3-GLuc (red) or tra [file pone.0053138.s001.pdf]

A

WT HIV

Cell

Supernatant

0ng 5ng 10ng 25ng 0ng 5ng 10ng 25ng

Pr55Gag-

p24(CA)-

42kD Actin-

anti-HA-

HA-Tethrin (ng)

0 5 10 25

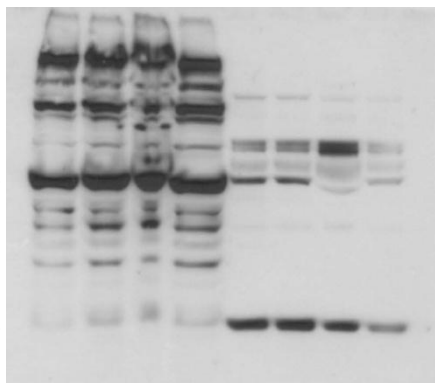

B

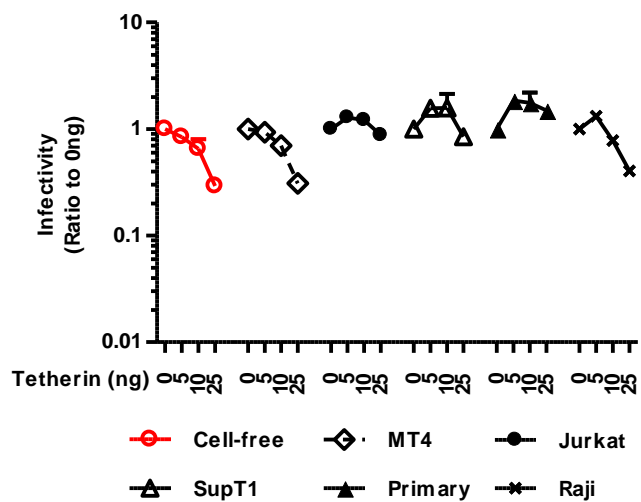

Supplement: Figure S2 — Control experiment for Figure 3B, C for HEK293 cells expressing tetherin and wild-type HIV. (A) A Western blot analysis as in Figure 3B for HEK293 cells expressing wild-type HIVLAI that does not lack Vpu and increasing amounts of tetherin (ng). (B) Relative HIVNL4-3-GLuc infectivity released by HEK293 producer cells expressing increasing amounts of tetherin (red) or transmitted from producer cells to indicated target cell types in co-cultures. The infectivity was normalized to non-tetherin expressing cells. Error bars represent the standard error of the mean from 3 experiments. (PDF) [file pone.0053138.s002.pdf]

## Analysis Cell Lines

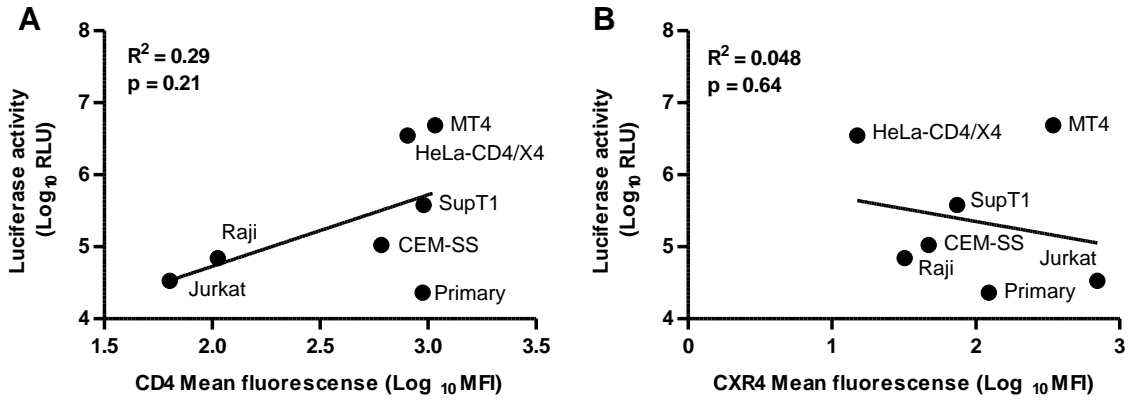

## Analysis Jurkat Clones

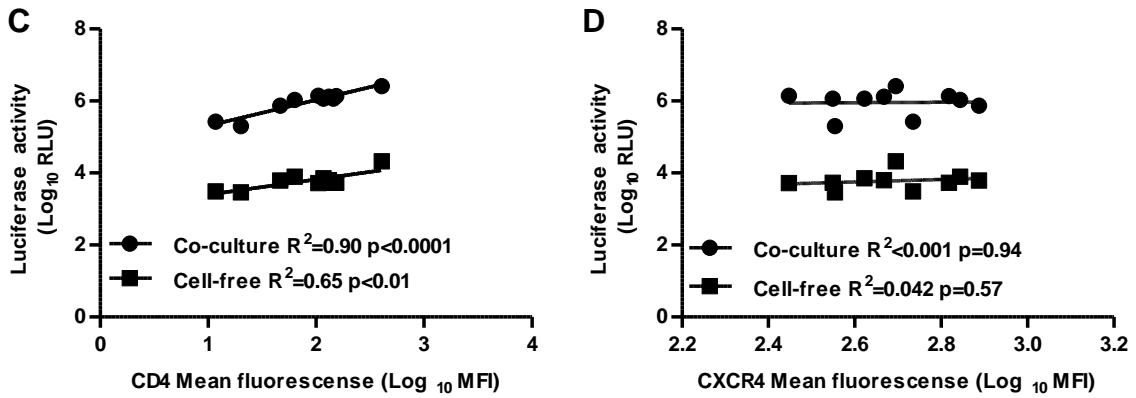

Supplement: Figure S3 — Susceptibility to HIV correlates weakly with CD4 expression among various cell types except in an isogenic background of Jurkat clones. (A, B) Indicated cell types were infected by spinoculation with concentrated cell-free HIVNL4-3-GLuc and luciferase activity was measured 36 h post-inoculation. Luciferase activity was correlated with CD4 and CXCR4 expression levels. R2 and p-values from a linear regression test are shown. (C, D) Individual Jurkat clones derived from Jurkat E6.1 displaying various levels of CD4 expression were spinoculated with concentrated HIVNL4-3-GLuc or co-cultured with HEK293 donor cells producing HIVNL4-3-GLuc. The resulting luciferase activity was measured 36 h post-inoculation. Luciferase activity was correlated with CD4 and CXCR4 expression levels. R2 and p-values from a linear regression test are shown. (PDF) [file pone.0053138.s003.pdf]

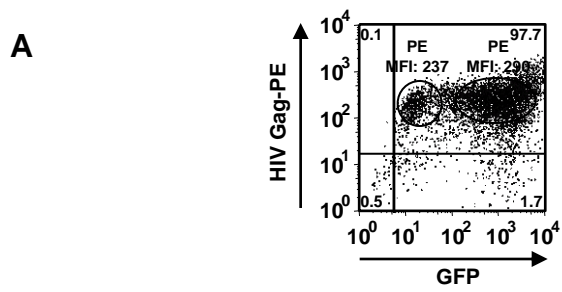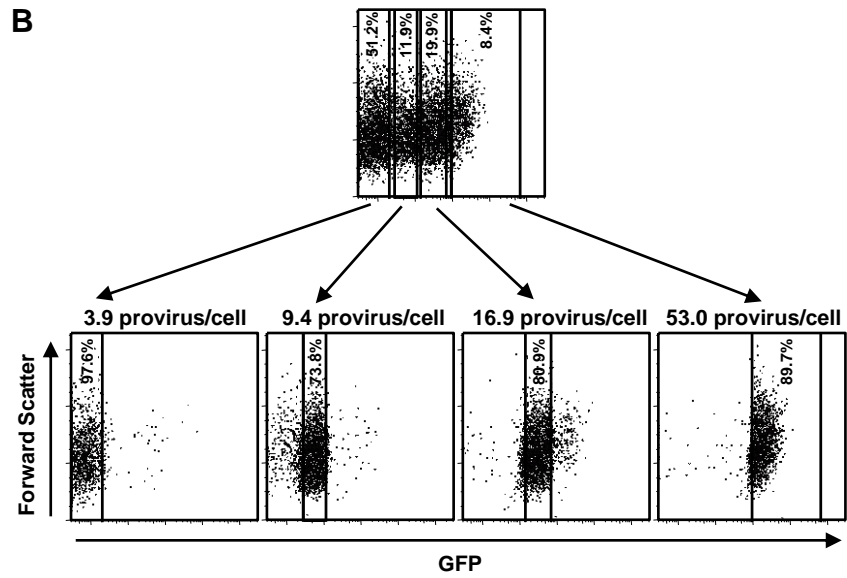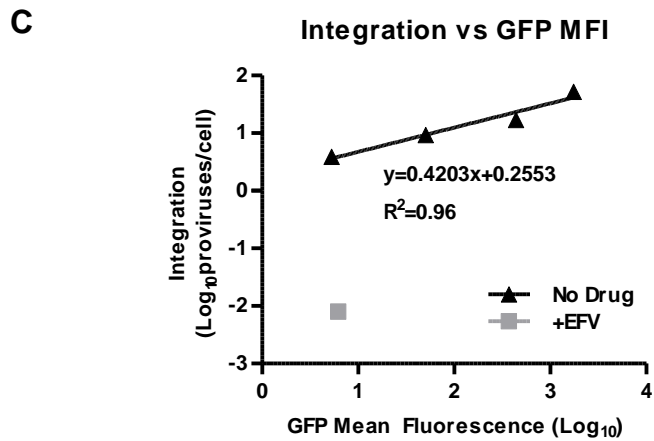

Supplement: Figure S4 — Experimental details for correlating GFP expression levels with the numbers of HIV proviruses shown in Figure 9B . (A) Comparison of HIV Gag fluorescence with GFP intensity. MT4 cells were infected with cell-free HIVIRES-GFP. HIV Gag (α-Gag-PE) and GFP expression (GFP) were measured 36 h post-infection. Mean fluorescence intensity of HIV Gag-PE was calculated for GFPlo and GFPhi populations. Figure illustrates that higher infection levels per cell are better detected from GFP fluorescence intensity than from HIV Gag-PE fluorescence intensity. (B) Jurkat cells were inoculated with concentrated HIVIRES-GFP (VSV-G) and incubated at 37°C for 24 h. Cells were then sorted into separate populations based on GFP mean fluorescence. Sort purity is indicated in the gates of each sorted population. HIV integration was measured from the sorted populations by Alu-PCR. The level of proviruses/cell is indicated above each population dot plot. (C) The number of proviruses/cell was correlated with the GFP mean fluorescence by linear regression. The limit of detection is at ∼9 proviruses/cell based on a sample treated with 1 µM efavirenz (EFV). (PDF) [file pone.0053138.s004.pdf]

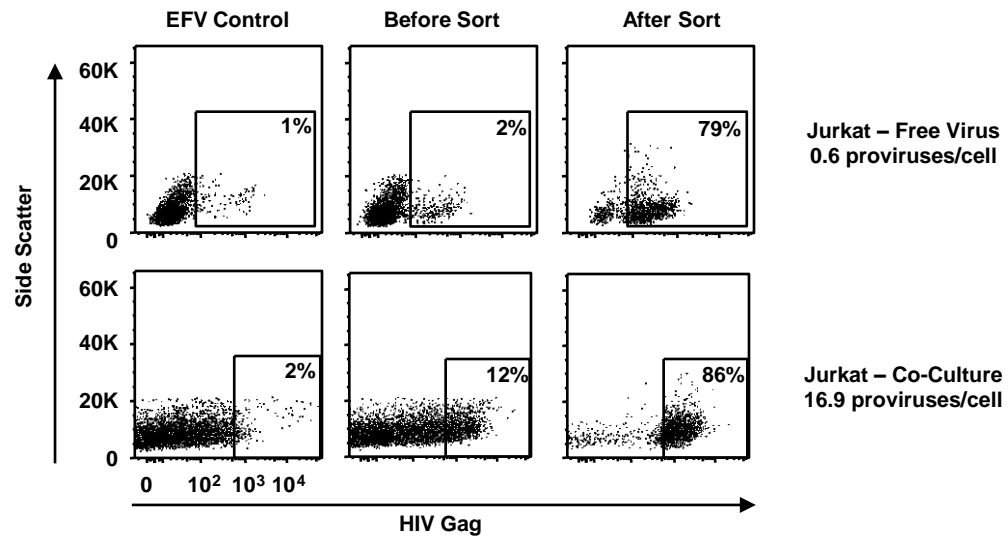

Supplement: Figure S5 — Experimental details for the determination of proviral numbers in cell-free infection or in co-cultures presented in Figure 9C . CFSE and HIV-Gag double positive Jurkat cells were sorted from cell-free infections or co-cultures. Sorting gates were set based on a 1 µM efavirenz (EFV)-treated control. “After sort” displays the purity of the sorted cell fraction. Note that percent infection shown under “Before Sort” column may not represent actual percentage of infected cells due to interfering staining coming from bound viral particles. The number of HIV integration events of these sorted cells was analyzed by Alu-PCR (see Figure 9C). (PDF) [file pone.0053138.s005.pdf]

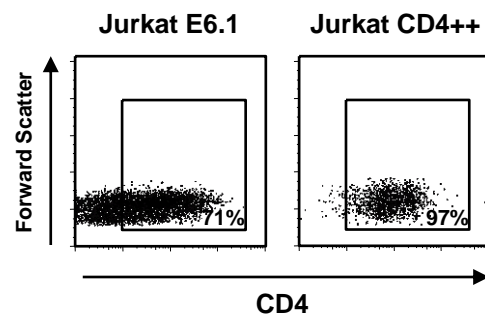

Supplement: Figure S6 — Generation of Jurkat E6.1 cells with high CD4 expression. Jurkat E6.1 cells introduced from ATTC displayed a larger portion of cells lacking CD4 expression. Cells were sorted for CD4 expression to generate a Jurkat E6.1 population that expressed CD4 more homogeneously (CD4++). All experiments in this study were conducted with this polyclonal Jurkat CD4++ population. (PDF) [file pone.0053138.s006.pdf]
